# The apical ECM preserves embryonic integrity and distributes mechanical stress during morphogenesis

**DOI:** 10.1242/dev.150383

**Published:** 2017-12-01

**Authors:** Thanh Thi Kim Vuong-Brender, Shashi Kumar Suman, Michel Labouesse

**Affiliations:** 1 Sorbonne Universités, UPMC Université Paris 06, CNRS, Laboratoire de Biologie du Développement - Institut de Biologie Paris Seine (LBD - IBPS), Paris 75005, France; 2 Development and Stem Cells Program, IGBMC, CNRS (UMR7104), INSERM (U964), Université de Strasbourg, 1 rue Laurent Fries, BP10142, Illkirch 67400, France

**Keywords:** Apical extracellular matrix, *C. elegans*, Embryonic elongation, Laser nano-ablation, Muscle anchoring, Zona pellucida protein

## Abstract

Epithelia are bound by both basal and apical extracellular matrices (ECM). Although the composition and function of the former have been intensively investigated, less is known about the latter. The embryonic sheath, the ECM apical to the *Caenorhabditis elegans* embryonic epidermis, has been suggested to promote elongation of the embryo. In an RNAi screen for the components of the sheath, we identified the zona pellucida domain proteins NOAH-1 and NOAH-2. We found that these proteins act in the same pathway, and in parallel to three other putative sheath proteins, the leucine-rich repeat proteins SYM-1, LET-4 and FBN-1/Fibrillin, to ensure embryonic integrity and promote elongation. Laser nano-ablation experiments to map the stress field show that NOAH-1 and NOAH-2, together with PAK-1/p21-activated kinase, maintain and relay the actomyosin-dependent stress generated within the lateral epidermis before muscles become active. Subsequently, loss-of-function experiments show that apical ECM proteins are essential for muscle anchoring and for relaying the mechanical input from muscle contractions, which are essential for elongation. Hence, the apical ECM contributes to morphogenesis by maintaining embryonic integrity and relaying mechanical stress.

## Abstract (precis)

**Summary:** In *C. elegans*, embryonic apical extracellular matrix acts as a protective layer, a rigid anchor for muscle function and transmit actomyosin forces to promote embryonic elongation.

## INTRODUCTION

The extracellular matrix (ECM) is a specialized structure providing mechanical support for tissue assembly and organ shape. It mainly consists of secreted or transmembrane fibrous proteins and polysaccharides, forming an organized meshwork closely associated with the cell membrane (Alberts et al., 2014). The ECM actively modulates various biological processes, such as cell proliferation, differentiation, migration and tissue morphogenesis by regulating biochemical and mechanosensitive signaling cues (Brown, 2011; Lu et al., 2011). Indeed, abnormal ECM composition and dynamics can induce congenital defects and diseases, including fibrosis and cancer (Naba et al., 2014; Lu et al., 2011). Extracellular matrices are present at both the basal and apical surfaces of epithelia. The role of the basal ECM has been widely explored, whereas less is known about the apical ECM (aECM) (Labouesse, 2012; Alberts et al., 2014). The composition of the aECM varies widely, from collagenous and chitinaceous cuticles of worms and insects, respectively (Page and Johnstone, 2007; Özturk-Çolak et al., 2016), to cellulose in plants (Cosgrove, 2005). As for the basal ECM, aECM components mediate numerous functions; they have been linked to fertility, hearing, renal and vascular diseases, cancers and morphogenesis (Jovine et al., 2005; Plaza et al., 2010). However, the mechanisms of aECM action are unclear.

**Fig. 1.**
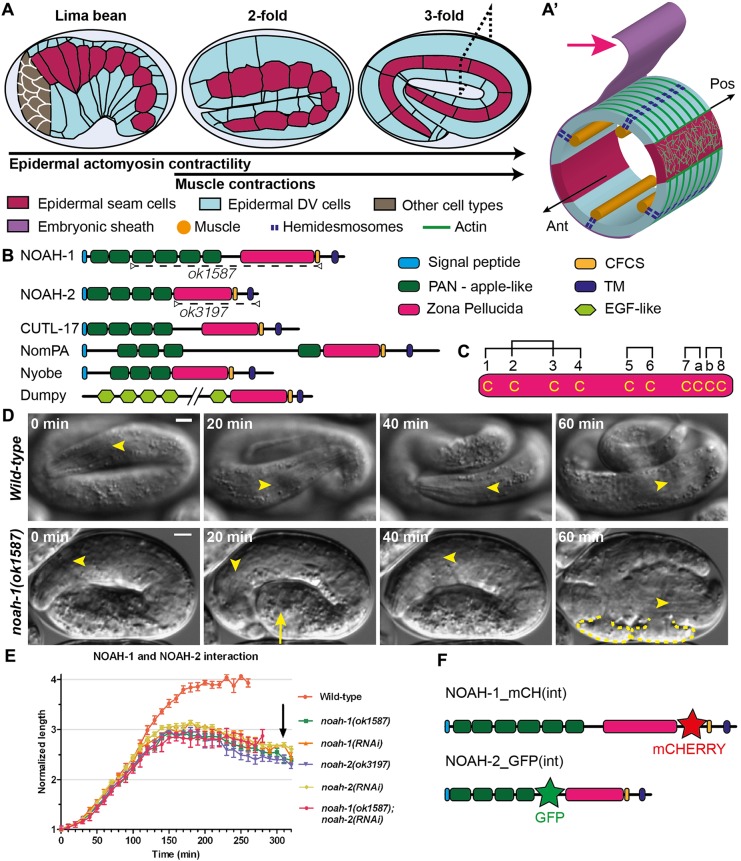
**Embryos defective for ZP-domain proteins NOAH-1 and NOAH-2 arrest elongation and rupture.** (A) Overview of *C. elegans* embryonic elongation: representative elongation stages [the relative (fold) increase in embryo length is used for staging] and epidermal cells are shown. Anterior to the left, dorsal up. (A′) Transverse section through the embryo (dashed rectangle in A); the gut, which occupies the inner cavity of the embryo, is not shown for simplicity. Red arrow, embryonic sheath; Ant, anterior; Pos, posterior. (B) SMART domain prediction for NOAH-1, NOAH-2 and related proteins; dashed lines indicate NOAH-1/2 domains predicted to be missing in *ok1587* and *ok3197* alleles. EGF, epidermal growth factor; TM, transmembrane. Note that only part of the large Dumpy protein is displayed. (C) NOAH-1/2 contain ten cysteines in the ZP domain. Brackets show possible disulfide bridges (Boja et al., 2003, Darie et al., 2004) (Fig. S1B). (D) Differential interference contrast time-lapse sequence of control and *noah-1(ok1587)* embryos, starting 5 h after ventral enclosure. Arrow indicates a bulge in the embryo; yellow dashed line, rupturing areas in the embryo (16/16 embryos examined); arrowheads, middle of the pharynx. Scale bars: 5 μm. (E) Embryonic elongation curves in different genetic backgrounds expressed as embryo length normalized to the initial length after ventral enclosure; mean and standard error (s.e.m.) are presented (*n*≥9 embryos for each genotype). The arrow indicates the approximate moment when *noah-1(RNAi)* and *noah-2(RNAi)* embryos ruptured. (F) Position of internal (int) fluorescent reporters in knock-in constructs used in this study (domains are labeled as in B).

Recent work has established the essential role of the aECM in shaping various organs, such as *Drosophila* wings, tracheal tube, and apical bristle-like structures called denticles, and the *C. elegans* excretory pore (Fernandes et al., 2010; Mancuso et al., 2012; Dong et al., 2014; Ray et al., 2015). To investigate the role of the aECM, we turned to *C. elegans* embryonic elongation, during which embryos increase their length fourfold along the antero-posterior (AP) axis (Fig. 1A,A′) (Priess and Hirsh, 1986). This process is powered by epidermal actomyosin contractility and muscle contractions (Vuong-Brender et al., 2016). The embryonic aECM, also called the embryonic sheath (ES), is laid just before the beginning of embryonic elongation, and might pre-pattern the larval aECM (the cuticle), which anchors muscles through a trans-epidermal adhesion structure related to hemidesmosomes (referred to as CeHDs) (Fig. 1A′) (Moerman and Williams, 2006; Pásti and Labouesse, 2014). Moreover, the ES might transmit the epidermal actomyosin tension during elongation, as it contacts the epidermis right above actin bundles (Priess and Hirsh, 1986). Furthermore, digestion of this layer with trypsin generates embryos with serious body deformation, suggesting that they are unable to withstand tension (Priess and Hirsh, 1986).

The composition of the aECM has not yet been systematically investigated. The leucine-rich repeat (LRR) proteins SYM-1, EGG-6 and LET-4, and the zona pellucida (ZP) domain protein FBN-1/Fibrillin have been proposed to be ES proteins, because they line the outer part of the embryo and/or are secreted in the extra-embryonic space. In addition, FBN-1 mediates pharynx attachment and SYM-1 might help attach muscles to the cuticle (Davies et al., 1999; Mancuso et al., 2012; Kelley et al., 2015). Single and some double mutant combinations for these genes display a less severe phenotype than that observed after sheath-trypsin digestion of the embryonic sheath, suggesting the existence of additional aECM proteins.

To investigate this possibility, we performed an RNAi screen for transmembrane or secreted proteins required for embryonic integrity and elongation. We thereby identified two members of the ZP domain protein family, NOAH-1 and NOAH-2. We created CRISPR knock-in functional fluorescent reporters of these proteins to investigate their localization. Combining genetic analysis and imaging, we examined how NOAH-1 and NOAH-2 interact with one another and with other putative sheath proteins to promote embryonic integrity and elongation. Finally, using laser nano-ablation, we investigated the mechanical properties of the ES and how it drives embryonic elongation in combination with the actin cortex. Our work suggests that an association of cellular components with distinct material properties, akin to a composite material, promotes tissue elongation.

## RESULTS

### RNAi screen for essential embryonic sheath proteins identifies NOAH-1 and NOAH-2

We identified potential ES proteins based on two assumptions: (1) they should be essential for embryonic integrity during elongation; (2) they are secreted or transmembraneous. Using Wormbase (www.wormbase.org), we extracted a list of genes reported to be embryonic lethal in the RNAi database (Gunsalus et al., 2004) and containing a signal peptide or transmembrane domain (TMD). We further excluded proteins with well-described functions and those not expressed in the epidermis (e.g. neuropeptides). The final list contained 53 genes (Table S1). We subsequently carried out an RNAi screen by feeding and looked for the presence of ruptured embryos during elongation. The gene *noah*-*1* and its paralog *noah-2* (additionally tested) were the only hits we identified (below we collectively refer to both paralogs as *noah-1/2*).

### The ZP proteins NOAH-1 and NOAH-2 are required for embryonic integrity and elongation

We decided to further characterize NOAH-1/2, which are related to the *Drosophila melanogaster* aECM component NompA. NompA is required for the attachment of mechanosensory dendrites to the cuticle, enabling flies to hear and coordinate their movements (Chung et al., 2001). Like NompA, NOAH-1/2 are predicted to contain a signal peptide, several PAN_AP (plasminogen, apple-like) domains, a ZP domain and a TMD followed by a short cytoplasmic domain (Fig. 1B). The ZP and TMD are separated by a tetrabasic motif corresponding to a consensus furin cleavage site (CFCS), indicating that NOAH-1/2 could be cleaved from the TMD. The ZP domain includes two subtypes based on their similarity with ZP1-3 proteins of the mammalian egg coat (Jovine et al., 2005), which differ in the number of cysteine residues predicted to form intra-domain disulfide bridges. The ZP domain of NOAH-1/2 contains ten cysteines, as in ZP1/ZP2, rather than eight cysteines as in ZP3 (Fig. 1C; Fig. S1). NOAH-1/2 are highly conserved within nematodes, especially among *Caenorhabditis* species (Fig. S1). In particular, BLAST (Basic Local Alignment Search Tool) searches identified the nematode non-collagenous insoluble cuticle components (cuticlins) CUTL-17/18/27 (Fig. 1B) (Fujimoto and Kanaya, 1973; Sebastiano et al., 1991). Interestingly, the ZP-containing cuticlins CUT-1 and CUT-(3-6) are essential for the formation of cuticle longitudinal ridges (termed alae) and body morphology (Sapio et al., 2005; Sebastiano et al., 1991). Other identified similar proteins include the *Drosophila* aECM proteins Nyobe, Neyo, Trynity, Morpheyus and Dumpy required for shaping embryonic denticles (Fernandes et al., 2010) (Fig. 1B; Fig. S1).

To confirm the RNAi phenotype and further examine the function of NOAH-1/2, we obtained the mutations *noah-1(ok1587)* and *noah-2(ok3197)* from the *C. elegans* Genetic Center (CGC). The allele *noah-1(ok1587)* deletes part of intron 2 up to most of exon 6; the 5′ splice site of intron 2 might be spliced together with the 3′ splice site of intron 6, resulting in an in-frame deletion of the last four PAN_AP domains and the entire ZP domain (Fig. 1B). It is likely to be a strong hypomorph or a null allele. The allele *noah-2(ok3197)* removes nearly the entire exon 4, leading to a frame shift, resulting in the deletion of ZP domain and the following C-terminal part; it should also be a strong hypomorph or null allele. Both mutations were embryonic lethal with a phenotype very similar to that observed after RNAi, i.e. embryos occasionally exhibited abnormal bulges, arrested after reaching the 3-fold stage, and ruptured thereafter (Fig. 1D,E; Movie 1; Tables S2 and S3).

In summary, NOAH-1/2 structurally resembled many ZP-domain aECM proteins, which are important for the generation of specialized cuticle appendages in *C. elegans* and *Drosophila*.

### NOAH-1 and NOAH-2 form a potential heterodimer localizing apically to the epidermis

To define the expression pattern of NOAH-1/2 and whether they are indeed ES components, we generated several translational fluorescent reporters. Extra-chromosomal C-terminal GFP fusions, denoted NOAH-1::GFP(Cter) and NOAH-2::GFP(Cter), could rescue the embryonic lethality of *noah-1(ok1587)* and *noah-2(ok3197)*, respectively, indicating that they are functional. Likewise, a CRISPR knock-in line of NOAH-1::GFP(Cter) behaved like wild type. A CRISPR knock-in NOAH-1::GFP(Cter) and an extra-chromosomal NOAH-2::GFP(Cter) both displayed a dotted pattern in the epidermis starting at the birth of epidermal cells (Fig. S2A-F).

**Fig. 2.**
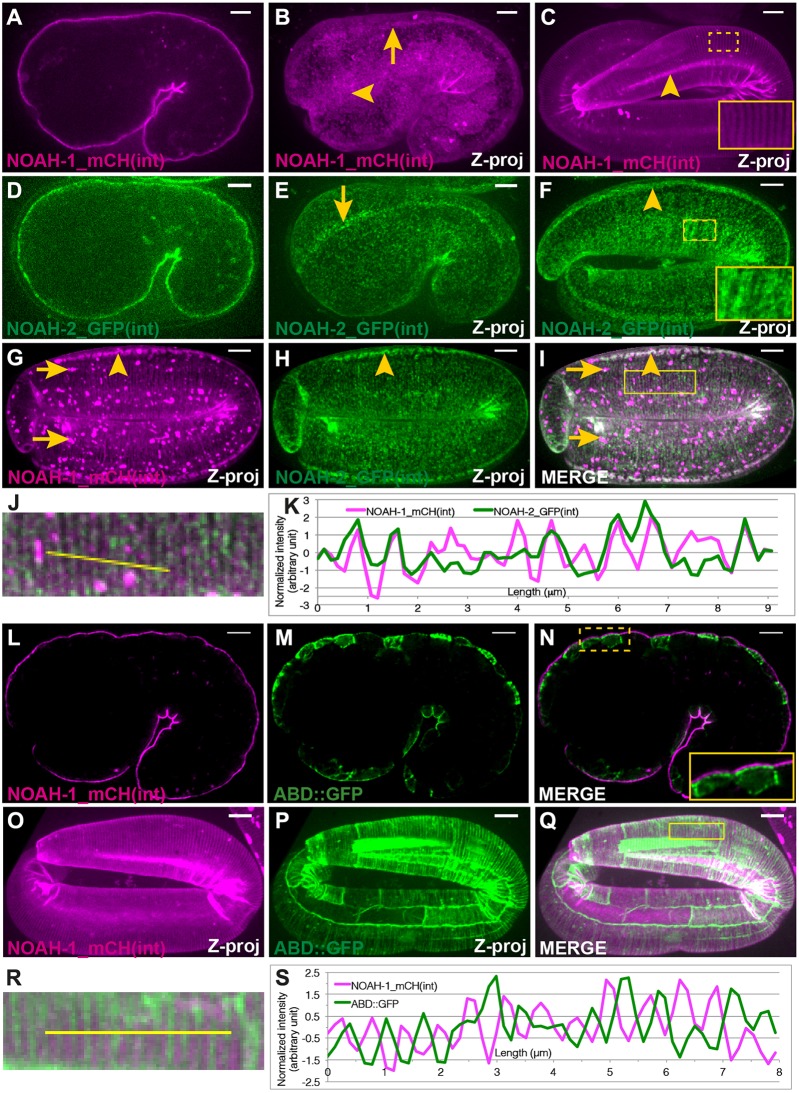
**NOAH-1 and NOAH-2 colocalize at the epidermal apex in areas located between actin bundles.** (A-F) Fluorescent images of NOAH-1_mCH(int) (A-C) and NOAH-2_GFP(int) (D-F) at the 1.5-fold (A,B,D,E) and pretzel (C,F) stages showing how they wrap around embryos. (A,D) Focal plane through the middle of the embryos; (B,C,E,F) *z*-projections (Z-proj). NOAH-1 and NOAH-2 were enriched at structures reminiscent of CeHD localization and pattern (arrows in B,E) and in seam cells (arrowheads in B,C,F; see O-Q and Fig. 3A′,A″ for seam cell position). (G-K) *z*-projection images of a 3-fold embryo carrying both NOAH-1_mCH (int) (G) and NOAH-2_GFP(int) (H); (I) merged image. NOAH-1 and NOAH-2 colocalized in seam cells (arrowheads). Note that the presence of NOAH-2_GFP(int) marker induced some aggregation of NOAH-1_mCH(int) (arrows) rarely seen in control (C). (J) Magnified view of the boxed area in I. (K) Normalized line profile of the yellow line in J. NOAH-1 peaks coincide with those of NOAH-2. (L-Q) Fluorescent images of embryos expressing NOAH-1_mCH(int) (L,O) and an actin-binding reporter (ABD::GFP; M,P) at the 1.5-fold (L-N; single focal plane through the middle) and 3-fold (O-Q; *z*-projection) stages. (N,Q) Merged images; inset in N shows that NOAH-1_mCH(int) localized apically to cortical actin. (R) Magnified view of the boxed area in Q. (S) Normalized line profile of the yellow line in R. NOAH-1 stripes alternate with actin stripes. Insets in C,F,N show higher magnifications of the boxed areas. Scale bars: 5 μm.

**Fig. 3.**
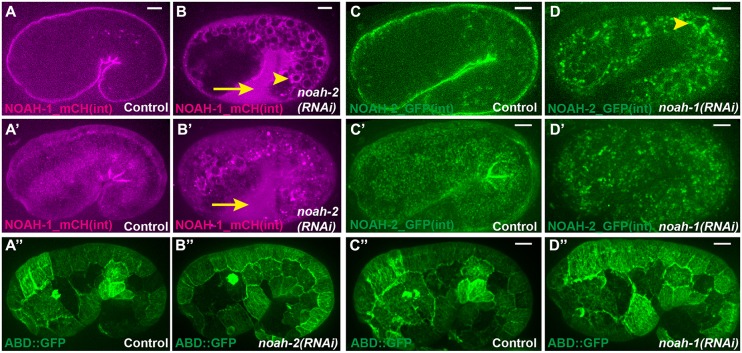
**NOAH-1 and NOAH-2 require each other for apical secretion.** (A-B″) Fluorescent images of control embryos carrying both NOAH-1_mCH(int) and ABD::GFP (A-A″), or the same strain treated with *noah-2(RNAi)* (B-B″). (C-D″) Fluorescent images of control embryos carrying NOAH-2_GFP(int) (C,C′) or ABD::GFP (C″) markers, and the same strains treated with *noah-1(RNAi)* (D-D″). The ABD::GFP expression showed some cell-to-cell variation. (A-D) Focal plane through the middle of the embryo; (A′-D″) *z*-projection. Note the perinuclear accumulation (arrowheads in B,D) of NOAH-1_mCH(int) (B) and NOAH-2_GFP(int) (D), and the extra-embryonic presence of NOAH-1_mCH(int) (arrows in B,B′); the actin pattern was not affected (A″-D″). Scale bars: 5 μm.

As some ZP-domain proteins are cleaved from the TMD at the CFCS (Jovine et al., 2005, Plaza et al., 2010), the dotty pattern of NOAH-1/2 described above might only reflect the localization of their C-terminal tail. To identify the localization of the N-terminal part, we built NOAH-1 and NOAH-2 reporters with a fluorescent protein inserted N-terminally to the CFCS (Fig. 1F), denoted NOAH-1_mCH(int) and NOAH-2_GFP(int), respectively. Homozygous NOAH-1_mCH(int) CRISPR-knock-in animals were healthy and behaved like wild type (Table S2), indicating that this construct is fully functional. Although an extra-chromosomal array expressing NOAH-2_GFP(int) rescued the embryonic lethality of *noah-2(ok3197)* and homozygous CRISPR-knock-in strain has little embryonic lethality (Table S2), the animals were generally sluggish, indicating that the construct might be only partially functional. These strains showed that NOAH-1 and NOAH-2 were distributed as a layer wrapping around the embryos with local differences (Fig. 2A-F), consistent with the notion that they are ECM components. Specifically, before the 2-fold stage, NOAH-1_mCH(int) and NOAH-2_GFP(int) were enriched above seam cells (Fig. 2B,C,F) and in lines positioned like CeHDs at that stage (Fig. 2B,E; see Fig. 3A′,A″ for epidermal cell identification with junctional actin) (Zhang et al., 2011). At later elongation stages, they displayed a fiber-like pattern in dorso-ventral (DV) cells (Fig. 2C,F, insets), consistent with ZP proteins, which are often organized in filaments (Jovine et al., 2005). NOAH-2_GFP(int) displayed a dottier pattern and less obvious circumferential stripes at the 3-fold stage, perhaps because it was only partially functional. Near hatching, NOAH-1_mCH(int) localization collapsed in big aggregates that persisted until adulthood (Fig. S2G-I).

When combined, NOAH-1_mCH(int) and NOAH-2_GFP(int) showed colocalization, in particular at the circumferential stripes at late elongation stages (Fig. 2G-K; Fig. S2J-L). As the circumferential stripes were reminiscent of the actin bundles in the DV epidermal cells, we compared NOAH-1 distribution with that of epidermal actin visualized with a GFP-labeled actin-binding domain (referred to as ABD::GFP) (Gally et al., 2009). Deconvoluted confocal images (Fig. 2L-N) and fluorescence profile measurement (Fig. S2M,N) clearly indicated that NOAH-1 was apical to cortical actin, consistent with an extracellular localization. Furthermore, merged images at late elongation showed that NOAH-1_mCH(int) stripes were enriched apical to the area separating actin bundles (Fig. 2O-S).

To define the epistasis relationship between *noah-1* and *noah-2*, we co-depleted both proteins. The resulting phenotype was similar to that in the single mutants (Fig. 1E), suggesting that *noah-1/2* act in the same pathway. Upon NOAH-2 RNAi-knockdown in a strain carrying both NOAH-1_mCH(int) and the ABD::GFP marker, NOAH-1_mCH(int) became abundant in the extra-embryonic space, and accumulated in intracellular structures surrounding the cell nucleus reminiscent of the endoplasmic reticulum (Fig. 3A-B′; Fig. S3). Likewise, in *noah-1(RNAi)* embryos, NOAH-2_GFP(int) also accumulated intracellularly, but not in the extra-embryonic space (Fig. 3C-D′; Fig. S3). By contrast, the actin pattern was not affected (Fig. 3A″-D″). We conclude that NOAH-1 and NOAH-2 required each other for their normal secretion.

Taken together, the different subcellular localization of internal and C-terminal NOAH-1/2 fluorescent reporters, the distribution of both proteins in circumferential stripes apically to actin, and the presence of NOAH-1_mCH(int) in the extra-embryonic space when NOAH-2 is depleted strongly suggest that NOAH-1 and NOAH-2 are cleaved from their membrane anchor and are ES components. Because NOAH-1 and NOAH-2 colocalized, we speculate that they physically interact with each other and form heterodimers, as previously observed for the zona pellucida ZP1/2 proteins of mouse eggs or VEα/VEβ proteins of the rainbow trout vitelline membrane (Boja et al., 2003; Darie et al., 2004).

### A network of extra-embryonic proteins redundantly ensure embryonic integrity and elongation

As mentioned earlier, the LRR proteins SYM-1, EGG-6 and LET-4, and the ZP-protein FBN-1, have also been suggested to be ES components (Davies et al., 1999; Mancuso et al., 2012; Kelley et al., 2015). Interestingly, *let-4*, which affects the aECM in the excretory pore region, is synthetic lethal with *sym-1* with a phenotype similar to that of NOAH-1/2 depletion. To define the possible functional relationships between these proteins and NOAH-1/2, we measured the elongation kinetics of double-deficient embryos. We excluded EGG-6, as its depletion by RNAi induces early embryonic lethality (Mancuso et al., 2012).

**Fig. 4.**
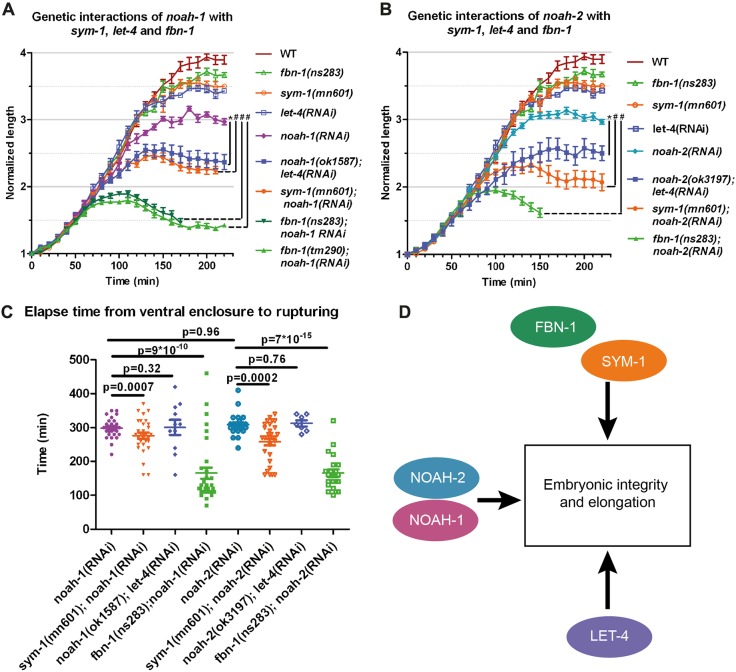
**NOAH-1/2 act in a parallel pathway to SYM-1, FBN-1 and LET-4.** (A,B) Embryonic elongation curves in different genetic backgrounds. *n*≥9 except for *noah-2(ok3197); let-4(RNAi)* embryos (*n*=7). (C) Time interval from ventral enclosure to the first rupturing signs in different genetic backgrounds. (D) Proposed scheme of NOAH-1/2, SYM-1, FBN-1 and LET-4 interactions. The mean and s.e.m. from experimental data are presented; *P*-values from two-tailed *t*-tests are shown. For A and B, *t*-test was performed using the embryo length at 150 min after ventral enclosure; *P<0.001, ^#^*P*<0.0001.

Homozygous *sym-1(mn601)* and *fbn-1(ns67, ns283, tm290)* embryos, as well as *let-4(RNAi)* embryos, elongated essentially like wild type but with a slightly shorter final length (Fig. 4A). *mn601* is considered null (Davies et al., 1999); *ns67* and *ns283* are point mutations probably resulting in loss of function, whereas *tm290* is a deletion resulting in larval arrest (Kelley et al., 2015). Depleting NOAH-1 in *sym-1(mn601)* or LET-4 in *noah-1(ok1587)* mutants induced a more severe phenotype, whereby embryos arrested between the 2-fold and 2.5-fold stages then ruptured (Fig. 4A). Moreover, treating *fbn-1* mutants with RNAi against *noah-1* led to embryonic arrest at the 1.8-fold to 2-fold stages [similarly to the embryos most severely affected by trypsin treatment as described by Priess and Hirsh (1986)], and shortly thereafter ruptured (Fig. 4C), with a penetrance varying from nearly 100% (*tm290* and *ns283*) to 54% *(ns67*; Fig. S4A; Table S3). Moreover, *sym-1; noah-1* and *fbn-1; noah-1*, but not *let-4; noah-1* defective embryos ruptured earlier than *noah-1(RNAi)* embryos, consistent with a stronger interaction (Fig. 4C). *noah-2* showed similar interactions with these mutants compared with *noah-1* (Fig. 4A-C). These data suggest that *noah-1/2* act in a pathway parallel to *sym-1*, *let-4* and *fbn-1.* Consistent with these results, we found that CRISPR knock-in SYM-1::GFP(Cter) (partly functional; Fig. S4B) was enriched at the DV/seam cell border and formed circumferential stripes colocalizing with NOAH-1_mCH(int) at late elongation (Fig. S5A-H). This pattern confirmed the previously described SYM-1 cuticular annuli localization in larvae (Davies et al., 1999). We could not generate internal CRISPR reporters for LET-4, EGG-6 or FBN-1, which all have a predicted TMD (SYM-1 lacks a predicted TMD), although we obtained a C-terminal fusion of LET-4 (Fig. S5I-N).

To determine whether *let-4*, *sym-1* and *fbn-1* act in a single pathway*,* we investigated genetic interactions between them*.* We found that *let-4; fbn-1* deficient embryos showed a synthetic 3-fold arrest and rupturing, similar to *sym-1; let-4* deficient embryos (Mancuso et al., 2012). The penetrance of *let-4* and *fbn-1* interaction depended on the *fbn-1* alleles, as observed for the *noah-1/2* and *fbn-1* interaction (Fig. S4A). Double *sym-1(mn601); fbn-1(ns283)* and *sym-1(mn601); fbn-1(tm290)* mutants elongated normally, suggesting that both genes acted in the same pathway in parallel to *let-4.* Collectively, the genetic interactions between NOAH-1/2, LET-4, SYM-1 and FBN-1 suggest that they act in three parallel pathways (Fig. 4D). As NOAH-1 and NOAH-2 have a similar role, we used either NOAH-1 or NOAH-2 depletion in subsequent experiments.

**Fig. 5.**
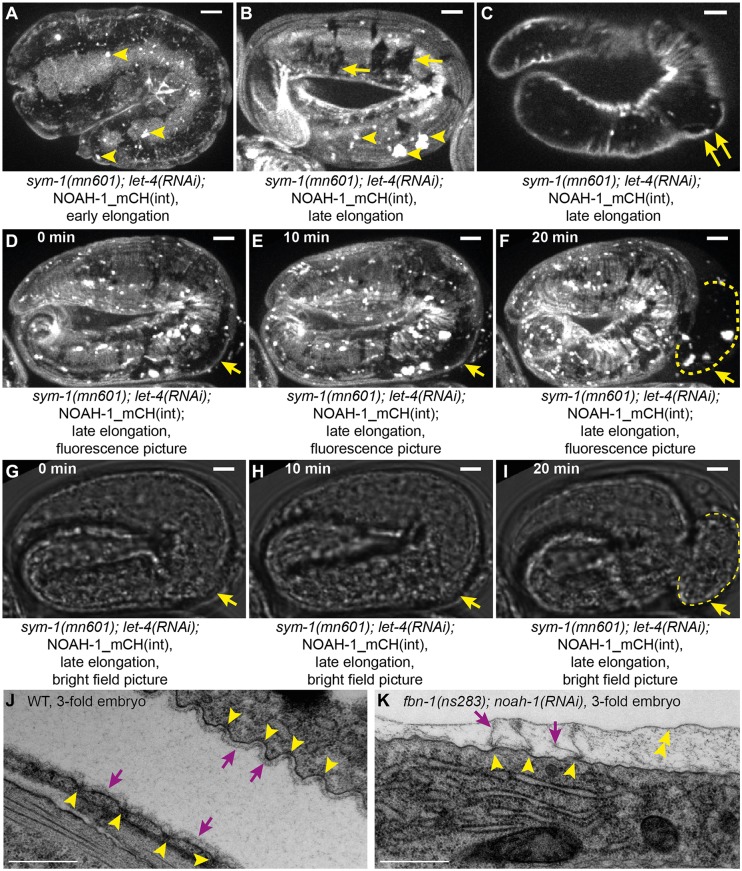
**Depletion of putative sheath proteins damages the embryonic sheath.** (A-I) NOAH-1_mCH(int); *sym-1(mn601); let-4(RNAi)* embryos at early (A) and late (B,C) elongation stages, and through a time-lapse sequence (D-I). The expression pattern showed some aggregates (arrowheads in A,B), and important lesions beyond the 2-fold stage (arrows in B). A bulge appeared coinciding with a damaged region (double arrows in C). (D-I) Fluorescence (D-F) and brightfield (G-I) images of the same embryo as shown in C arresting at the 3-fold stage; the damaged area (arrows) increased in size prior to embryo rupturing (dashed line). (A,B,D-F) *z*-projection; (C,G-I) single focal planes through the middle of the embryo; *n*=15/15. Scale bars: 5 μm. (J,K) Electron micrographs of wild-type (J) and *fbn-1(ns283); noah-1(RNAi)* (K) embryos at the equivalent to 3- to 3.5-fold stage. Arrowheads indicate attachment points of the embryonic sheath to the plasma membrane; arrows indicate embryonic sheath, which is continuous in controls but disrupted in *fbn-1(ns283); noah-1(RNAi)* embryos; double arrowhead points to a layer likely to be the permeability layer reported by Olson et al. (2012). Scale bars: 500 nm. *n*=3 for both genotypes.

To define the cellular basis of the rupturing phenotype, we examined the ES in *sym-1(mn601); let-4(RNAi);* NOAH-1_mCH(int) embryos. Before the 2-fold stage, NOAH-1_mCH(int) localization in *sym-1(mn601); let-4(RNAi)* formed small aggregates more frequently than did wild type (Fig. 5A; Fig. 2B). At later stages, we observed areas lacking NOAH-1_mCH(int) labeling in both DV and seam epidermal cells, which could correspond to ES damaged regions (Fig. 5B), as they often coincided with bulging out areas (Fig. 5C). These defects, except for the bulges, were also observed in homozygous *sym-1(mn601)* or *let-4(RNAi)* embryos, but were less severe (Fig. S6). The size of the damaged regions increased during elongation until the embryos eventually ruptured (Fig. 5D-I). Because embryonic rupture can be due to myosin II hypertension as in *mel-11/MYPT* or *rga-*2/*RhoGAP* mutants, which is best observed as adherens junction defects (Diogon et al., 2007), we examined the junctional marker AJM-1::GFP in *noah-1(ok1587)* mutants, but did not see any irregularity before rupturing (data not shown). We conclude that NOAH-1 depletion did not cause embryonic rupture by increasing tension on junctions.

To assess the nature of the physical damage on the ES, we used electron microscopy in *fbn-1(ns283); noah-1(RNAi)* embryos, which had the most severe elongation and earlier integrity defects. We examined late elongation embryos (corresponding to a normal 3- to 3.5-fold stage, based on elongation timing), when the actin bundles have acquired a regular spacing. In wild-type embryos, the ES was seen as a thin layer apical to the epidermal apical plasma membrane, with regularly spaced anchor points (Fig. 5J), which have been proposed to coincide with the actin bundle position (Priess and Hirsh, 1986). In *fbn-1(ns283); noah-1(RNAi)* embryos, we could still identify the ES attached to anchor points, but it was discontinuous in between (Fig. 5K). Owing to the weak staining of the ES and the presence of extra-embryonic material of unknown identity, in some wild-type (11/40) and mutant (10/36) representative images, we could not conclude on the continuity of the ES; however, clear disruption was found in most of the mutant (22/36) as opposed to only one (out of 40) of the wild-type images. Thus, the area where the ES was damaged in *fbn-1(ns283); noah-1(RNAi)* embryos corresponds to where NOAH-1 and SYM-1 (SYM-1 acting in the same pathway as FBN-1) were enriched in late embryos (Fig. 2O-S; Fig. 4D; Fig. S5A-H).

Altogether, we conclude that the LRR proteins SYM-1 and LET-4, together with the ZP proteins NOAH-1/2 and FBN-1 define three parallel pathways acting to preserve embryonic integrity. Their absence strongly affects ES continuity.

### Depletion of sheath proteins disrupts hemidesmosomes and muscle function

As detailed above, embryos lacking sheath proteins ruptured at least an hour after elongation arrest (Fig. 4A-C). Thus, integrity problems cannot account for elongation defects. While measuring embryonic growth kinetics (Fig. 4), we noticed that *fbn-1(ns283); noah-1(RNAi)* embryos failed to move properly. We previously showed by time-lapse video microscopy that actin filaments are laterally displaced by muscle contractions (Zhang et al., 2011). By imaging epidermal actin filaments and muscle nuclei, we found that *fbn-1(ns283); noah-1(RNAi)* embryos exhibited less pronounced twitching and never rolled within the eggshell, like wild type do, indicating that muscle function was indeed strongly affected (Movies 2, 3). As embryos with abnormal muscles (Williams and Waterston, 1994) or defective CeHDs involved in anchoring muscles to the cuticle (Zhang and Labouesse, 2010) arrest elongation at or before the 2-fold stage, the failure of *fbn-1(ns283); noah-1(RNAi)* embryos to move could account for their arrest at that stage.

**Fig. 6.**
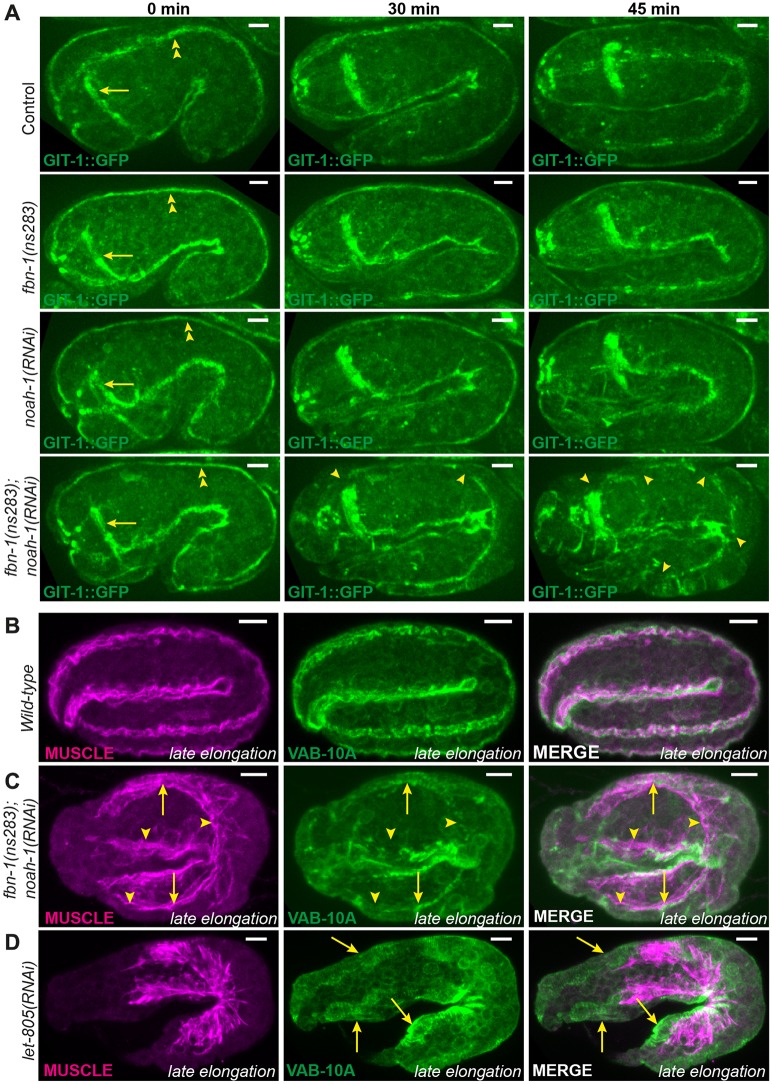
**Embryonic sheath protein depletion induces hemidesmosome disruption and muscle detachment.** (A) GIT-1::GFP fluorescence time-lapse sequences showing discontinuous CeHDs in an *fbn-1(ns283); noah-1(RNAi)* embryo beyond the 2-fold stage (*n*=16/16), which was not observed in *fbn-1(ns283)* (*n*=14/14)*, noah-1(RNAi)* (*n*=6/6) or control (*n*=6/6) embryos. Arrows indicate nerve ring; double arrowheads, CeHDs; single arrowheads, regions of disrupted CeHDs. (B-D) Wild-type (B), *fbn-1(ns283); noah-1(RNAi)* (C) and *let-805(RNAi)* (D) embryos at late elongation stages stained with antibodies against a muscle-specific antigen and the CeHD component VAB-10A, and their merged images. Arrowheads in C indicate regions of disrupted CeHDs. Arrows in C,D indicate regions of CeHDs overlapping with muscle in *fbn-1(ns283); noah-1(RNAi)* but not in *let-805(RNAi)* embryos; *n*≥16. All panels are z-projections. Scale bars: 5 μm.

Given its position, the ES might anchor muscles through CeHDs, much like the cuticle in larvae, and thereby transmit muscle tension to the whole body. To see whether muscles were properly anchored in *fbn-1(ns283); noah-1(RNAi)* embryos, we examined the localization of two hemidesmosomal markers, GIT-1 and VAB-10A (Bosher et al., 2003; Zhang et al., 2011), and of muscles. Before the 2-fold stage, *fbn-1(ns283); noah-1(RNAi)*; GIT-1::GFP embryos showed longitudinal lines corresponding to CeHDs, as observed in control (Fig. 6A, 0 min). However, as embryos were reaching the 2-fold stage, GIT-1::GFP displayed discontinuities in *fbn-1(ns283); noah-1(RNAi)* (Fig. 6A, 30 min), which became more pronounced later on (Fig. 6A, 45 min). We did not observe these defects in single *fbn-1(ns283)*, *noah-1(RNAi)* or control embryos (Fig. 6A; Fig. S7). Consistently, *fbn-1(ns283); noah-1(RNAi)* embryos double-stained for VAB-10A and muscles showed no defects compared with wild-type embryos before the 2-fold stage (data not shown), but exhibited enlarged, disorganized muscle quadrants and interrupted VAB-10A pattern after the 2-fold stage (Fig. 6B,C). Importantly, however, the remaining VAB-10A still partially overlapped with myofilaments, suggesting that muscles and CeHDs pulled off from the outer edge of the embryo together, and that they had detached from the apical side of the epidermis. This was in contrast to the depletion of the CeHDs receptor LET-805 (myotactin) at the basal side of the epidermis (Hresko et al., 1999), where myofilaments are disorganized and detach from the epidermis, but VAB-10A staining remained at the outer edge of embryos instead of being juxtaposed to muscles (Fig. 6D). Therefore, NOAH-1 and FBN-1 act at the epidermis apex, corroborating the hypothesis that they are components of the ES. To determine whether *noah-1(RNAi)* embryos also exhibit hemidesmosomal defects before rupturing, we used time-lapse microscopy of endogenous VAB-10A::mCHERRY embryos, which shows stronger fluorescence at later stages. Out of the ten *noah-1(RNAi)* embryos imaged only one showed CeHD defects before rupturing (Fig. S8). In particular, *noah-1(RNAi)* embryos could actively move and roll within the eggshell.

To see whether muscle contractions also influence ES remodeling as has been shown for CeHDs (Zhang et al., 2011), we looked at the localization of NOAH-1 when muscle contraction is disrupted. In embryos treated with RNAi against *unc-112*/Kindlin (Rogalski et al., 2000), NOAH-1_mCH(int) circumferential stripes were absent or their formation was delayed (Fig. S9); moreover, 8/40 embryos ruptured and holes in the ES were observed (Fig. S9). Holes in the ES structure were also seen in 9/27 β-integrin defective *pat-3(st564)* mutant embryos which also have no muscle contraction (Williams and Waterston, 1994) (Fig. S9). Thus, the absence of muscle contractions affected the integrity of the ES and its remodeling in a process suggestive of a positive feedback.

In conclusion, depletion of aECM proteins affected not only embryonic integrity but also muscle anchoring and function, supporting the notion that ES performs the same task as the cuticle in larvae. The loss of muscle anchoring largely accounted for the elongation arrest observed when NOAH-1 and FBN-1 are defective.

### The embryonic sheath relays the actomyosin stress and preserves its anisotropy

The results described above suggest that the closely apposed ES and actin bundles relay the stress produced by muscles. To determine whether the ES directly relays epidermal actomyosin stress, we turned to a stage when muscles are not yet active, making it possible to assay mechanical stress using physical methods.

**Fig. 7.**
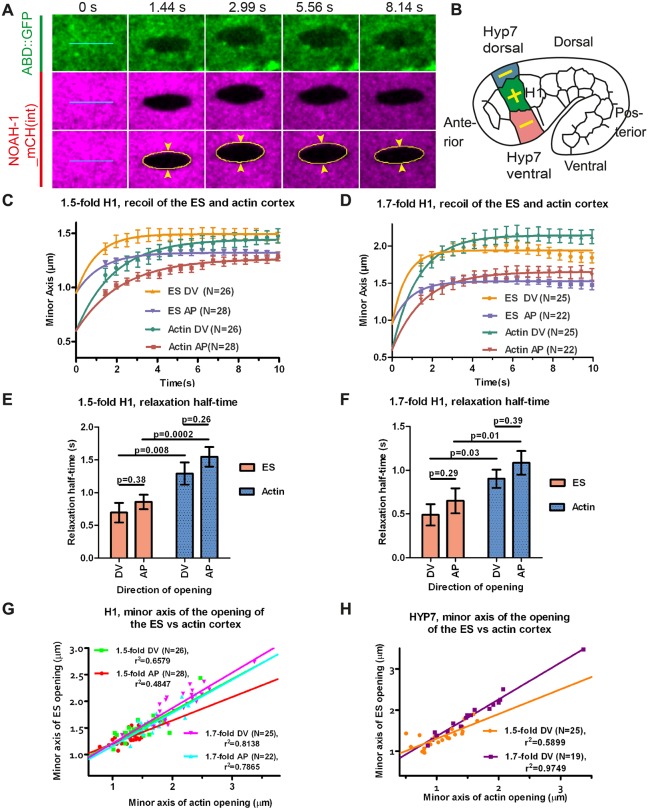
**The embryonic sheath transmits actomyosin stress.** (A) Time-lapse video micrographs before (0 s) and after laser ablation (1.44 s is the first image after ablation) of the actin cortex and the ES in the seam cell H1 of a 1.7-fold embryo. Cyan lines, 5 µm-long laser cuts; yellow line, elliptical fit of the cut opening (only shown for the ES); arrowheads, distance between the cut borders (minor axis of the opening ellipse). (B) Ablations were performed in the H1 cell (along the AP and DV directions, yellow cross) and in its dorso-ventral HYP7 neighbors (along the AP direction, yellow lines). (C-F) Plot and single exponential fit of the minor axis of the cut opening versus time (C,D) and relaxation half-time (E,F) for the embryonic sheath and the actin cortex at the 1.5-fold (C,E) and 1.7-fold (D,F) stages for the seam cell H1 (see Eqns 2 and 3 in Materials and Methods). *P*-values obtained from Z-test. (G,H) Minor axis of the opening at equilibrium of the embryonic sheath plotted against that of the actin cortex for the cell H1 (G) and the cell HYP7 (H) derived from curve fitting in C and D. Solid lines, linear fit with r^2^ values are shown; N, number of ablated embryos. DV and AP indicate directions perpendicular to the cut direction (AP and DV, respectively). The mean and s.e.m. from experimental data (C,D) and curve fitting (E,F) are presented.

First, we used laser nano-ablation to cut both the sheath and actin cortex layers [visualized with NOAH-1_mCH(int) and ABD::GFP markers]. We compared their recoil dynamics and the opening shape of the cut. The time that the cut borders take to reach their equilibrium position indicates the viscosity of the medium over the stiffness (Mayer et al., 2010), whereas the distance between the cut borders (minor axis of the opening ellipse) is proportional to ratio of mechanical stress in the direction perpendicular to the cut over layer stiffness (Fig. 7A) (Vuong-Brender et al., 2017). The stiffness reflects the resistance of an elastic material to deformation, whereas the viscosity describes the resistance of a fluid to flow. Altogether, this approach gives information on material properties and mechanical stress. We performed ablations in the head seam cell H1 and in its dorso-ventral cell neighbors (future HYP7), two representatives of epidermal cell types with distinct mechanical properties. Specifically, we induced 5 µm-long ablations along the AP or DV directions, then derived the mechanical stress in the direction perpendicular to that line from the minor axis of the opening ellipse (cut opening) (Vuong-Brender et al., 2017); for clarity, we will refer to the response to the cut by the direction perpendicular to the cut direction (Fig. 7B). We observed simultaneous opening of the ES and the actin cortex, indicating that both layers were cut together (Fig. 7A; Movie 4).

In the head seam cell H1, we found that the ES relaxed independently of the actin cortex (Fig. 7C,D), and with a significantly smaller relaxation half-time at both the 1.5-fold and 1.7-fold stages (Fig. 7E,F). We inferred that either the ES is more rigid or it relaxed in an environment that was less viscous than the actin cortex. As the actin cytoskeleton is known to be visco-elastic (Amblard et al., 1996; Tharmann et al., 2007), it is likely to relax in a more viscous medium. The H1 cortex and the ES above have isotropic material properties at those stages, as they exhibited a similar relaxation half-time along the AP and DV directions. Plotting the minor axis of the cut opening of the ES versus that of the actin cortex showed the relationship between the mechanical stresses on the two entities (Fig. 7G,H). As we observed a linear relationship between them, it indicates that the mechanical stress on the ES and actin cortex was linearly related at those elongation stages. Thus, the ES and the actin cortex may be physically linked in seam cells, but not tightly since they relaxed independently after laser ablation.

**Fig. 8.**
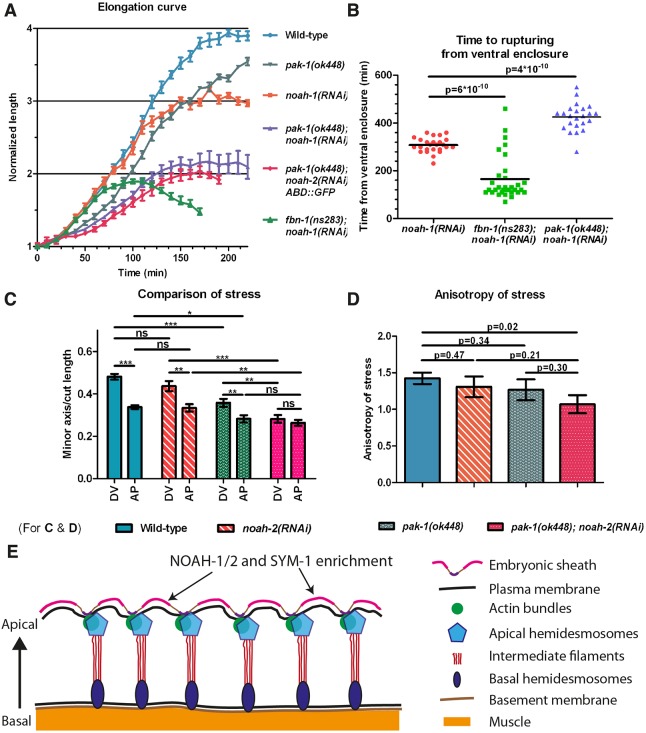
***pak-1* and *noah-1/2* cooperate to maintain stress anisotropy during embryonic elongation.** (A) Elongation curves in different genetic backgrounds; mean and s.e.m. is presented, *n*≥9. (B) Time interval from ventral enclosure to the moment that embryo rupturing signs were first observed for different genetic backgrounds. Note that all *pak-1(ok448)* embryos hatch without rupturing (*n*=51/51). (C) Ratio of the minor axis opening over cut length for the seam cell H1 at a stage equivalent to 1.7-fold for different genetic backgrounds. The s.e.m. was obtained from curve fitting. *P*-values obtained from two-tailed *t*-test. ns, not significant; *P<0.05, **P<0.01, ***P<0.001. DV and AP, directions perpendicular to the cut directions. (D) Anisotropy of stress and s.e.m. calculated from the data shown in C; *P*-values obtained from Z-test. (E) Diagram showing the hypothetical position of NOAH-1/2 and SYM-1 (FBN-1) sheath proteins; the areas of enrichment were drawn approximately.

To test whether the ES relays epidermal actomyosin tension, we examined the effects of mutants defective for non-muscle myosin II activity on *noah-1-*induced rupturing and elongation phenotypes. Two upstream activators of myosin II, LET-502/Rho Kinase and PAK-1, act in parallel to regulate *C. elegans* embryonic elongation (Gally et al., 2009). Specifically, thermosensitive *let-502(sb118ts)* mutants arrest elongation at the 2-fold stage when raised at 25.5°C, whereas *pak-1(ok448)* embryos hatch with a slightly shorter length; by contrast, *let-502(sb118ts); pak-1(ok448)* double mutants do not elongate at all at 25.5°C (Gally et al., 2009). We found that *let-502(sb118ts); noah-1(RNAi)* embryos at 25.5°C still ruptured after arresting at the 2-fold stage. However, *let-502(sb118ts); pak-1(ok448); noah-1(RNAi)* embryos at 25.5°C did not rupture up to 450 min after ventral enclosure (*n*=18/18; Movie 5). Thus, the mechanical stress on the ES depended at least in part on actomyosin. We also found that *pak-1(ok448); noah-1/2(RNAi)* embryos not only elongated at a slower rate but also arrested with a shorter length (at the 2-fold stage) compared with single *pak-1(ok448)* or *noah-1/2(RNAi)* embryos (Fig. 8A)*. pak-1(ok448); noah-1(RNAi)* embryos ruptured later than *noah-1(RNAi)* embryos, indicating that some part of stress on the sheath had been relieved (Fig. 8B). This was in contrast to *fbn-1(ns283); noah-1(RNAi)* embryos, which arrested with a length similar to that of *pak-1(ok448); noah-1(RNAi)* embryos but ruptured earlier, consistent with more severe damage on the ES as described previously. Thus, *pak-1(ok448); noah-1/2(RNAi)* had a more severe elongation phenotype but milder integrity defects. Notably, these embryos still had muscle contractions and rolled within the eggshell (Movie 6).

To determine whether the poor elongation of PAK-1 and NOAH-1/2 defective embryos was due to lower actomyosin stress, we used again laser nano-ablation on the actin cortex in the H1 cell at a stage equivalent to 1.7-fold (for staging, see Materials and Methods). We measured the ratio of minor axis opening to the cut length as a readout to compare the actomyosin stress (Vuong-Brender et al., 2017). We found that the stress in wild-type, *noah-2(RNAi)* and *pak-1(ok448)* embryos was higher in the DV direction compared with the AP direction (hence anisotropic), whereas it was nearly isotropic in *pak-1(ok448); noah-2(RNAi)* embryos (Fig. 8C,D). Although we could not detect statistically significant differences for the DV/AP stress ratio between single and double mutants, there was a clear difference comparing control with double mutant stress ratios (Fig. 8D). Combined with our recent results suggesting that a DV/AP stress ratio higher than 1.0 promotes *C. elegans* embryonic elongation (Vuong-Brender et al., 2017), the nearly isotropic stress observed in *pak-1(ok448); noah-2(RNAi)* embryos can account for their slower elongation and shorter final length (Fig. 8A,D).

In summary, our data carry two implications: first that the ES has different mechanical properties compared with the actin cortex, and second that the ES can relay the mechanical stress provided by actomyosin and the muscles.

## DISCUSSION

The aECM present in developing embryos has long remained a poorly characterized entity at the molecular and functional levels. Here, we undertook a systematic approach to identify proteins belonging to the ES, the aECM of *C. elegans* embryos. Thereby, we identified two ZP domain proteins, NOAH-1 and NOAH-2, as essential components of the ES. Their study demonstrated the role of the ES as a protective enveloping layer, as an essential muscle attachment site and as a mediator of both epidermal actomyosin- and muscle-induced stress.

Previous work on vertebrate and invertebrate ZP domain proteins has highlighted their role as a structural aECM component or as mechanotransducers (Jovine et al., 2005). NOAH-1/2 combine both a structural role, similar to their related proteins in *Drosophila* (Trynity, Neyo, Nyobe, Morpheyus), and a mechanical stress transmission function, as for *Drosophila* NompA (Plaza et al., 2010; Özturk-Çolak et al., 2016; Fernandes et al., 2010; Chung et al., 2001). Several lines of evidence strongly suggest that NOAH-1 and NOAH-2 are ES components that potentially form co-secreted heterodimers. First, they were colocalized apically to actin bundles and their secretion depended on each other. Second, loss of NOAH-1 and FBN-1 disrupted the continuity of the ES, and induced muscles and CeHD detachment together from the apical surface. The pattern of NOAH-1/2, which changed from a relatively homogeneous distribution to fiber-like circumferential stripes, is typical of other ZP domain proteins (Jovine et al., 2005). Interestingly, ECM circumferential fibrils can be found in other elongating structures, such as the chitin filaments present in the *Drosophila* tracheal tube (Özturk-Çolak et al., 2016) or the collagen fibrils found around *Drosophila* oocytes (Haigo and Bilder, 2011). These filaments might establish a molecular corset restricting expansion along the circumference and may subsequently pattern the worm cuticle annuli.

The ECM usually contains several classes of proteins working together to build the matrix. We found that NOAH-1/2 cooperated with another ZP protein, FBN-1, and two LRR proteins, SYM-1 and LET-4, to protect the embryo from mechanical damage. In the absence of sheath proteins, the embryos ruptured probably as a result of actomyosin-dependent tension during embryo elongation. The high level of redundancy explains the mild phenotype of mutants for only one sheath protein. The localization of NOAH-1/2 and SYM-1 between actin bundles, and the sheath disruption at the same position when NOAH-1 and FBN-1 are absent, suggest that NOAH-1/2, SYM-1 and FBN-1 strengthen the sheath mostly between actin bundles in DV epidermal cells (Fig. 8E). We did not attempt to identify the cellular receptors anchoring ES components to the plasma membrane and possibly actin bundles, but can speculate that the CeHDs proteins MUA-3 and MUP-4 could act as such receptors.

In addition to defects in embryonic integrity, depletion of sheath proteins induced elongation arrest. At least two factors contribute to this phenotype. First, embryonic sheath-defective embryos had very weak muscle contractions due to muscle detachment, which should prevent further elongation as in CeHD-defective embryos (Zhang and Labouesse, 2010). Thus, we propose that the ES provides a rigid anchoring point necessary for muscle function during embryogenesis. Likewise, the presence of a rigid extracellular anchorage helps the *Drosophila* wing resist the contraction of the hinge region during morphogenesis (Ray et al., 2015). Second, laser nano-ablation combined with genetic analysis have suggested that the ES can relay actomyosin tension. Indeed, we found that the mechanical stress exerted on the ES and the actin cortex were linearly related. Moreover, the rupturing phenotype of *noah-1* defective embryos was suppressed by removing actomyosin stress. Lastly, embryos deficient for *noah-1/2* and *pak-1* decreased the actomyosin stress anisotropy, resulting in reduced elongation rate and arrest at the 2-fold stage. Taken together, given the role of PAK-1 in promoting elongation in parallel to LET-502 (Gally et al., 2009), and our recent findings showing that stress anisotropy promotes elongation before muscle contractions (Vuong-Brender et al., 2017), we propose that the ES acts as a supracellular structure transmitting mechanical stress from one cell to another or from a particular location to the whole embryo.

In conclusion, even though our list of sheath proteins may not be exhaustive, our work constitutes a major step towards the understanding of ES function. By combining physical and genetic methods we established how the ES helps transmit mechanical stress, and it will be interesting to define how it contributes to shape the anisotropy of stress with PAK-1. Together, cortical actin and the ES form a composite material with significantly different physical properties in terms of rigidity and stress transmission.

## MATERIALS AND METHODS

### *C. elegans* alleles and strains

The Bristol strain N2 was used as the wild-type strain and nematodes were cultured as described by Brenner (1974). The strains used are listed in Table S4. The rescuing ability of plasmid constructs was checked by injecting them into the balanced *noah-1(ok1587)* and *noah-2(ok3197)* mutant strains, then looking for viable transgenic animals that did not segregate the balancers.

CRISPR knock-ins were either carried out as described elsewhere (Dickinson et al., 2013, 2015) or using a modified protocol with an asymmetric repair template (1.5 kb and 0.5 kb homologous arms) and single worm PCR detection.

### RNA interference

The RNAi screen was performed using the Ahringer-MRC feeding RNA interference library (Source BioScience, Nottingham, UK) and feeding protocol (Kamath et al., 2003). Other RNAi experiments were done using injection of double-stranded RNA synthesized from PCR-amplified genomic fragments using a T3 or T7 mMESSAGE mMACHINE Kit (Ambion).

### Search for homologous proteins and protein domain prediction

Potential homologs were identified using BLAST (NIH, MD, USA) with a cut-off E-value of 10^−4^. Protein domain prediction was obtained using SMART (EMBL, Heidelberg, Germany).

### Immunostaining and image acquisition

Indirect immunofluorescence for VAB-10A [primary: 4F2 (IGBMC, Strasbourg, France, 1/1000), secondary: FITC-conjugated] and muscle [primary: NE8/4C6 (MRC LMB - Cambridge Collection, UK, 1/100), secondary: Alexa647-conjugated] was as described elsewhere (Bosher et al., 2003). Stacks of images (30-35 confocal sections, 0.3 µm step size) were captured using a confocal Leica SP5 microscope and projected using ImageJ (Fiji) software (NIH, Bethesda, Maryland, USA; http://rsb.info.nih.gov/ij/).

### DIC time-lapse and elongation curve quantification

Embryos were mounted and imaged at 20°C, or at 25.5°C for *let-502(sb118ts)* mutants, and the embryo length was quantified as described elsewhere (Vuong-Brender et al., 2017).

### Spinning-disk microscopy

Fluorescent images of live embryos were acquired using a Roper Scientific spinning disk system (Zeiss Axio Observer.Z1 microscope, Yokogawa CSUX1-A1 confocal head, camera Evolve EMCCD 512×512 pixel, Metamorph software) with a 100× oil-immersion objective, NA=1.4. *z*-stacks of 0.3 µm step size were projected around the first 10 µm. Fluorescence time-lapses [*z*-stacks of the whole embryo (35 µm, 1 µm step size)] were made using a 63× oil-immersion objective, NA=1.4.

### Correction of chromatic shift for fluorescence colocalization experiments

We used images of 0.2 µm fluorescent microspheres (TetraSpeck, Life Technologies) and a custom ImageJ macro to calculate the red-green fluorescence shift, then applied inverse-shift for images used in Fig. 2 and Fig. S5D-H using MATLAB R2014b (The MathWorks).

### Deconvolution of confocal images

Images for Fig. 2L-N and for Fig. S2M-N were acquired using a Leica SP5 with a 63× oil-immersion objective, NA=1.4. *z*-stacks of seven focal planes of 0.2 µm step size were acquired and deconvoluted using the Huygens Essential software (Scientific Volume Imaging, Hilversum, The Netherlands).

### Calculation of line profile and normalization

For the comparisons shown in Fig. 2K,S and Fig. S5H, line intensity profiles were normalized using the following formula:
(1)
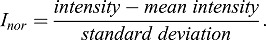


### Transmission electron microscopy

Embryos were high-pressure frozen and freeze-substituted as previously described (Weber et al., 2012). The samples were flat-embedded in Epon (Agar scientific) and 70 nm-thick sections (UC6, Leica Microsystems) were collected on formvar/carbon slot grids. Sections were then post-stained with 2% uranyl acetate and lead citrate. Samples were observed in a Tecnai12 (FEI, The Netherlands) TEM at 80 kV equipped with a 1K KeenView camera (Olympus).

### Laser ablation

Embryos of equivalent developmental stage determined by developmental timing were used. Laser cuts (length of 5 µm) and image analysis were performed as described elsewhere (Vuong-Brender et al., 2017). Curve fitting was performed with GraphPad Prism 5.00 using the following equation:
(2)
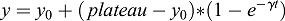
where y_0_ is the initial cut width, *plateau* is the minor axis of the opening at equilibrium and γ is the relaxation rate. *y*_0_=0.6 *μm* was determined elsewhere for actin cut (Vuong-Brender et al., 2017), *y*_0_=0.95 *μm* was estimated for the ES using photobleaching. The half-time of relaxation was defined as:
(3)
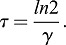


### Statistical analysis

For Fig. 8C, two-tailed *t*-tests were performed on the average of the last five time points (from about 8 s to 10 s) of the relaxation time course (Fig. 7D) using MATLAB. *z*-tests were performed using QuickCalcs (GraphPad Prism).

## Supplementary Material

Supplementary information

Supplementary information
