# Supplementary material for: The apical ECM preserves embryonic integrity and distributes mechanical stress during morphogenesis
Source: Development. 2017 Dec 1;144(23):4336–49. doi: 10.1242/dev.150383 (PMC5769628; doi:10.1242/dev.150383)
Supplement: Supplementary information [file develop-144-150383-s1.pdf]

Supplementary information

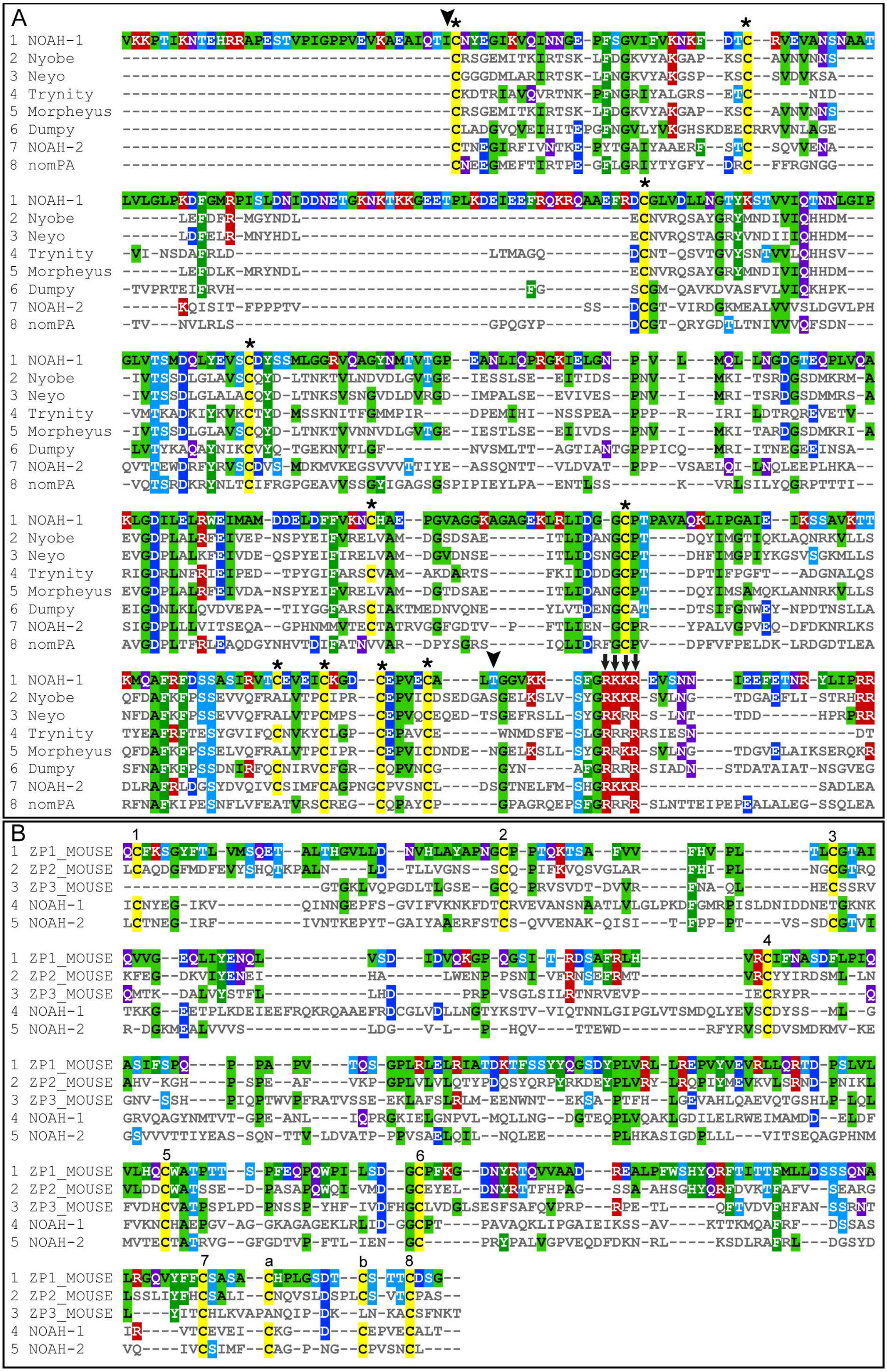

Fig. S1: Alignment of the ZP domain of NOAH-1 and NOAH-2 with other related proteins. (**A**) Alignment of NOAH-1 and NOAH-2 with similar proteins found with BLAST. The percentage of identity between NOAH- 1 and other *Drosophila melanogaster* homologues was determined using ClustalW (UniProt entry number in parenthesis): Nyobe (Q9V9X0), 23.3%; Trynity (Q8MS37), 23.9%; Morpheyus (Q9V9X1), 22%; Neyo (Q9VAG2), 20.8%; Dumpy (M9PB31), 20.2%; NOAH-2, 23.4%. It is comparable with the percentage of identity between cuticlin-like proteins and NOAH-1: CUTL-17 (Q20167, 26.4%), CUTL-18 (G5EBR9, 21.2%) and CULT-27 (Q18298, 20.9%), but smaller than the similarity between *C. elegans* NOAH-1 and its homolog from other nematode species: *C. remainei* (E3LY63, 96.3%), *C. brenneri* (G0NLF9, 96.0%), *C. briggsae* (A8WVZ7, 92.3%), *P. pacificus* (A0A0F5C4Z2, 67.0%), *S. ratti* (A0A090LAM2, 57.6%), *B. malayi* (A0A0K0J6N4, 56.3%). Black arrowheads show the beginning and end of the ZP domain; stars, conserved cysteines; red arrows, tetrabasic motif which is conserved among the proteins examined. The position of the ZP domain of NOAH-1 was predicted using SMART (**B**) Alignment of NOAH-1 and NOAH-2 with mouse ZP proteins ZP1/ZP2/ZP3. Numbers 1-8 and letters a-b denotes the cysteine number as described in (Jovine, 2005). The color code is as follows: dark-gray, mismatch; green, hydrophobic; yellow, cysteine; bright-blue, negative charge; bright-red, positive charge; dull-blue, alcohol; purple, polar. The alignment was performed using Kalign (EMBL, Heidelberg) and viewed using MView (EMBL, Heidelberg).

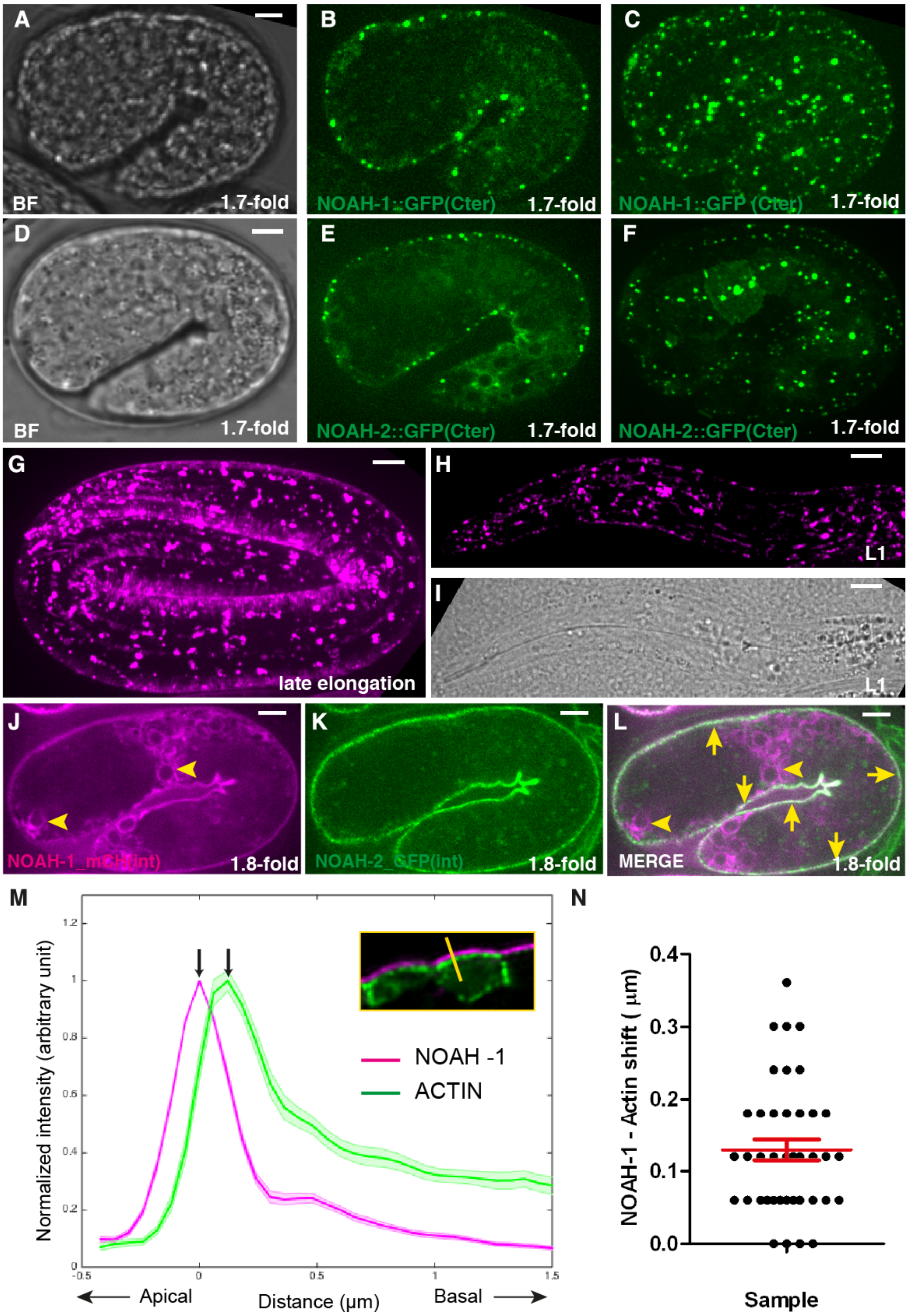

Development • Supplementary information

Fig. S2: (**A-F**) **Distribution of NOAH-1/2 C-terminal fusions**. Fluorescent images of a C-terminal GFP fusion with NOAH-1 (CRISPR knock-in, **A-C**) and NOAH-2 (extrachromosomal array, **D-F**) at the 1.7-fold stage. Note the dotted fluorescent pattern. (**ABDE**) single focal plane through the middle of the embryo; (**CF**) Z-projections. (**G-I**) **NOAH-1 forms aggregates in late elongation**. Fluorescent images of NOAH-1_mCH(int) at the end of elongation (**G**) or in the first larval stage (**H**); note the aggregates. (**I**) bright-field picture of the larvae shown in (**H**). (**J-L**) Fluorescent images of a typical 1.8-fold embryo carrying both NOAH-1_mCH(int) (**J**) and NOAH-2_GFP(int) (**K**); merged picture (**L**). Arrows show areas of colocalization between both markers. NOAH-2_GFP(int) construct induced some intracellular accumulation of NOAH-1_mCH(int) (arrowheads) not present in control embryos (see Fig. 2). (**MN**) **NOAH-1 is more apical compared to the actin cortex**. (**M**) Average line profile drawn along the apical-basal axis going through NOAH-1 and cortical actin (insert) visualized with the NOAH-1_mCH(int) and ABD::GFP markers; N=9 embryos, 4 line profiles per embryo; shading, standard error. (**N**) Spatial shift between actin and NOAH-1 peaks (marked with arrows in **M**), positive values correspond to more apical localization of NOAH-1. Z-test comparison with 0, $p=10^{-19}$. Line intensity profiles were drawn from outside to inside for each embryo, normalized to the maximum, then was shifted so that NOAH-1_mCH(int) intensity peaks coincided before doing the average and normalization again using MATLAB. Given the resolution of light imaging, the shift between the peaks gave only an indication of the respective position between NOAH-1 and actin, but not the precise distance between them. BF, bright field; scale bars, 5 μm.

Development • Supplementary information

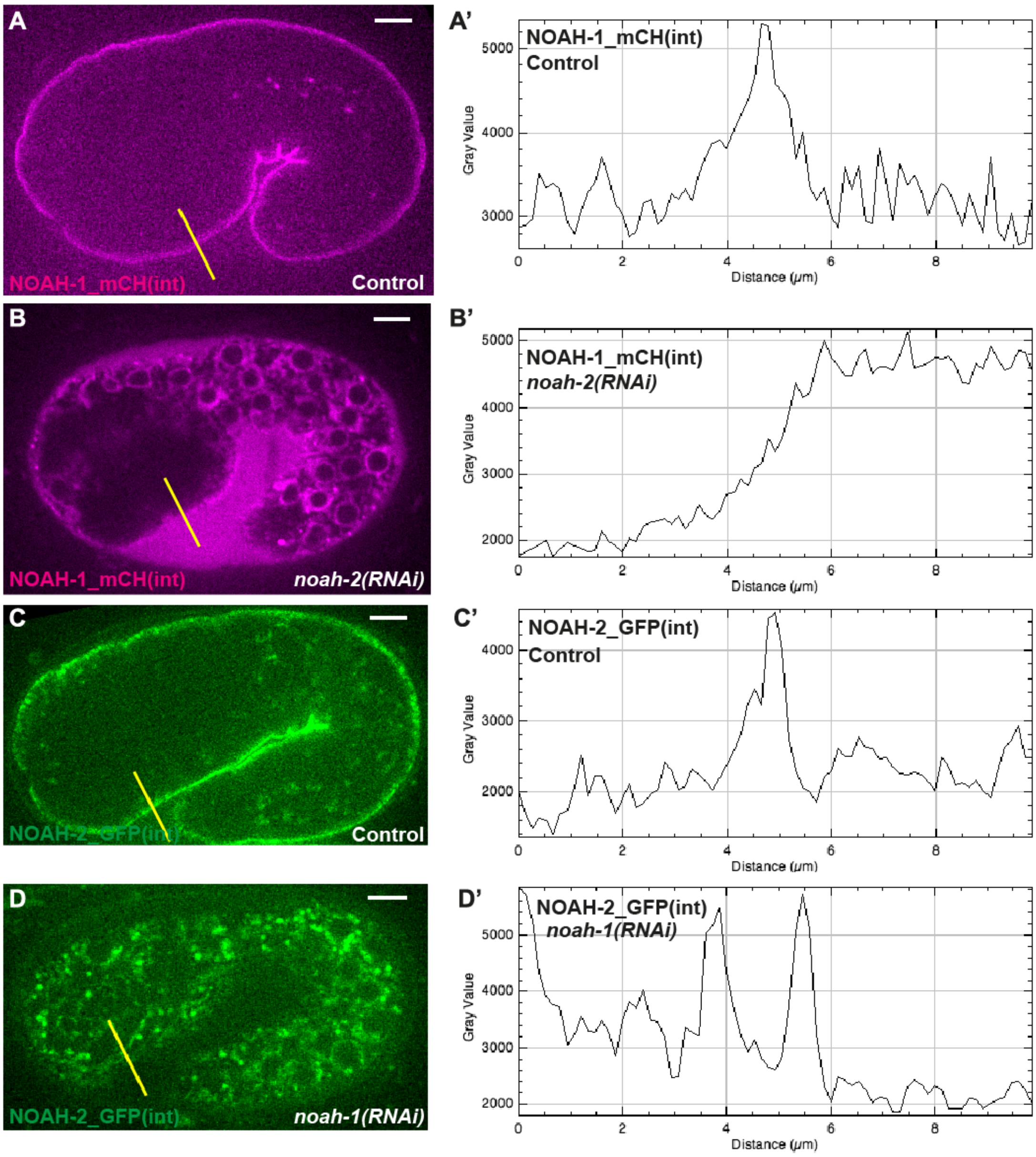

Fig. S3: NOAH-1 and NOAH-2 are mutually required for their proper localization. (**A**, **A'**, **B**, **B'**) Fluorescence pictures and line profiles (yellow lines, 3 pixels in width) of NOAH-1_mCH(int) localization in control (**A**, **A'**) and *noah-2(RNAi)* (**B**, **B'**) embryos. (**C**, **C'**, **D**, **D'**) Fluorescence pictures and line profiles (yellow lines, 3 pixels in width) of NOAH-2_GFP(int) localization in control (**C**, **C'**) and *noah-1(RNAi)* (**D**, **D'**) embryos. Line profiles were drawn from the inside to the outside of the embryos.

Development • Supplementary information

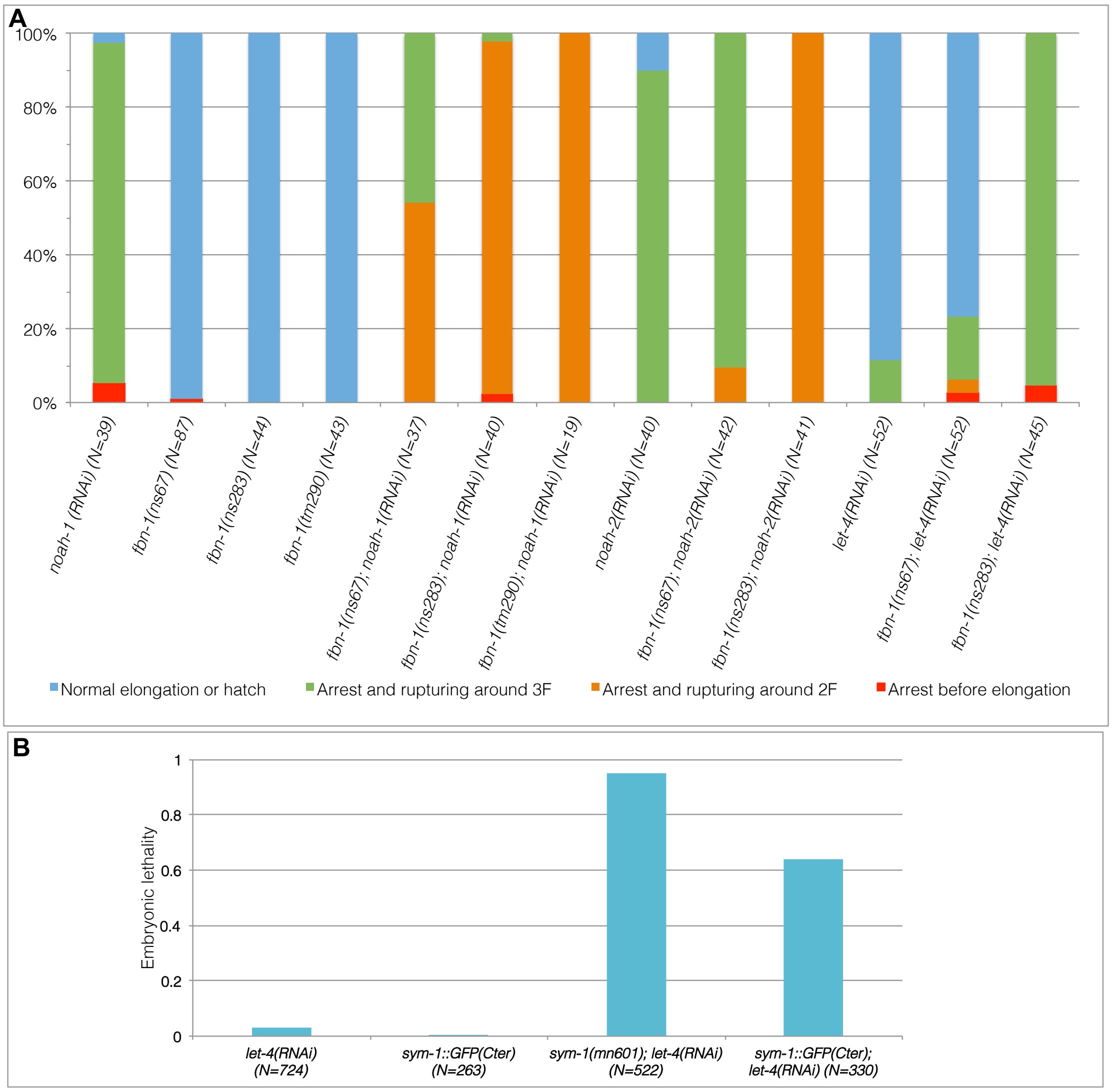

Fig. S4: (**A**) **Genetic interactions between *noah-1*, *noah-2*, *let-4* and different *fbn-1* alleles**. The terminal phenotypes of different genotypes were scored using time-lapse DIC movies; N, number of embryo examined. (**B**) **The CRISPR-knockin C-terminal GFP fusion of SYM-1 is partially functional.** The generated SYM-1::GFP(Cter) strain is homozygous viable but has a 64% penetrance synthetic lethality with *let-4(RNAi). let-4(RNAi)* had little embryonic lethality but induced 95% embryonic lethality in the *sym-1(mn601)* background. Thus CRISPR SYM-1::GFP(Cter); *let-4(RNAi)* embryos still had embryonic lethality, but lower than in the *sym-1(mn601)*; *let-4(RNAi)* background. The raw data is shown in table S3.

Development • Supplementary information

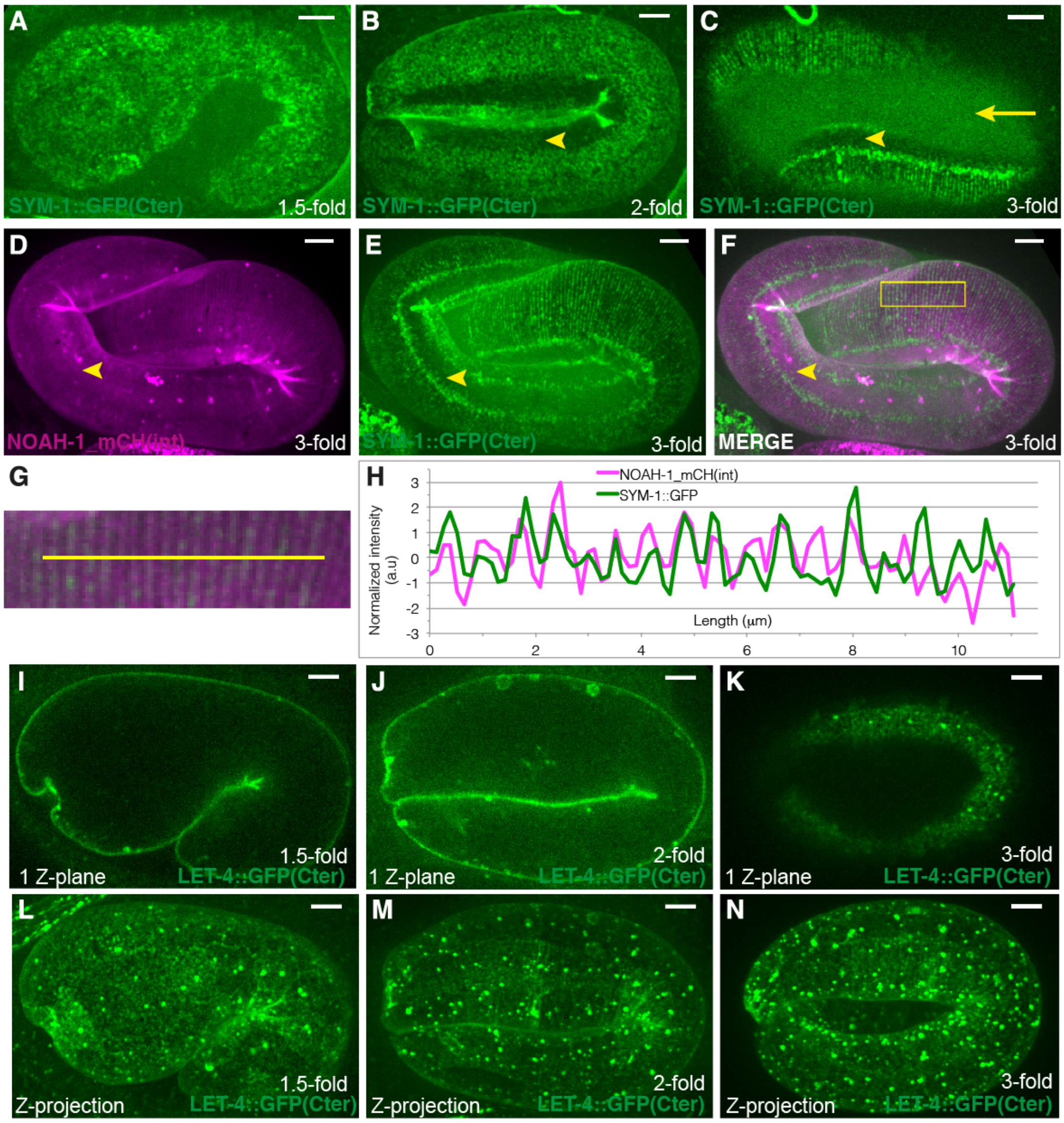

Fig. S5. (**A-H**) **SYM-1 circumferential parallel stripes colocalize with NOAH-1 stripes during late elongation**. (**A-C**) Fluorescence images of embryos homozygous for the CRISPR SYM-1::GFP(Cter) construct at the 1.5-fold, 2-fold and 3-fold stages, respectively. SYM-1::GFP(Cter) was present at a low level in the seam cells (arrowheads), and was found in the extra-embryonic space (arrow) at late elongation stages. The fact that SYM-1::GFP(Cter) is partly functional may account for the presence of SYM-1::GFP in the extra-embryonic space. (**D-H**) Fluorescence images of an embryo homozygous for both NOAH-1_mCH(int) (**D**) and SYM-1::GFP(Cter) (**E**) at around 3-fold stage; (**F**) merged image; (**G**) magnified view of the yellow box in (**F**); (**H**) line profile of the yellow line in (**G**) showing that NOAH-1 fluorescent intensity peaks coincide with SYM-1 peaks. (**I-N**) **Localization of a C-**

Development • Supplementary information

terminal LET-4 reporter (LET-4::GFP(Cter)) during embryonic elongation. Fluorescence images showing the localization of CRISPR knock-in LET-4::GFP(Cter) at the 1.5-fold, 2-fold and 3-fold stages for one focal plan through the middle of the embryo (**I,J**), and at the apex of the epidermis (**K**) and Z-projection (**L-N**), respectively. Scale bars, 5 μm.

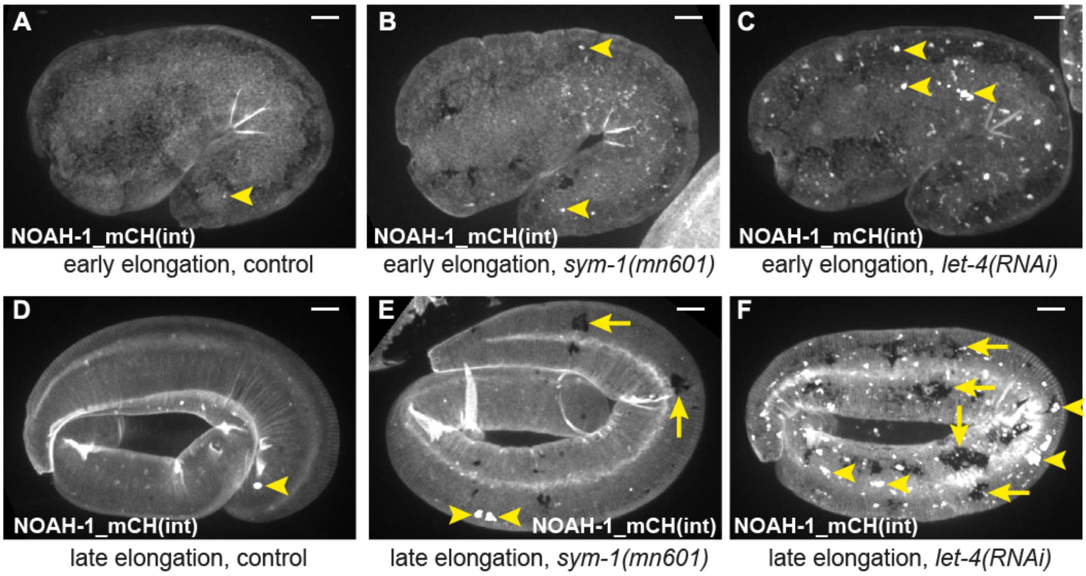

Fig. S6: The embryonic sheath is damaged in *sym-1(mn601)* mutant and *let-4(RNAi)* embryos. Embryos expressing NOAH-1_mCH(int) in control (**A**, **D**), *sym-1(mn601)* mutant (**B, E**) and *let-4(RNAi)* (**C, F**) embryos at early elongation (**A-C**) and late elongation (**D-F**) stages. Arrowheads show aggregation of NOAH-1_mCH(int); arrows show areas devoid of NOAH-1 fluorescence found in *sym-1(mn601)* mutant (**E**) and *let-4(RNAi)* (**F**) at late elongation stages.

Development • Supplementary information

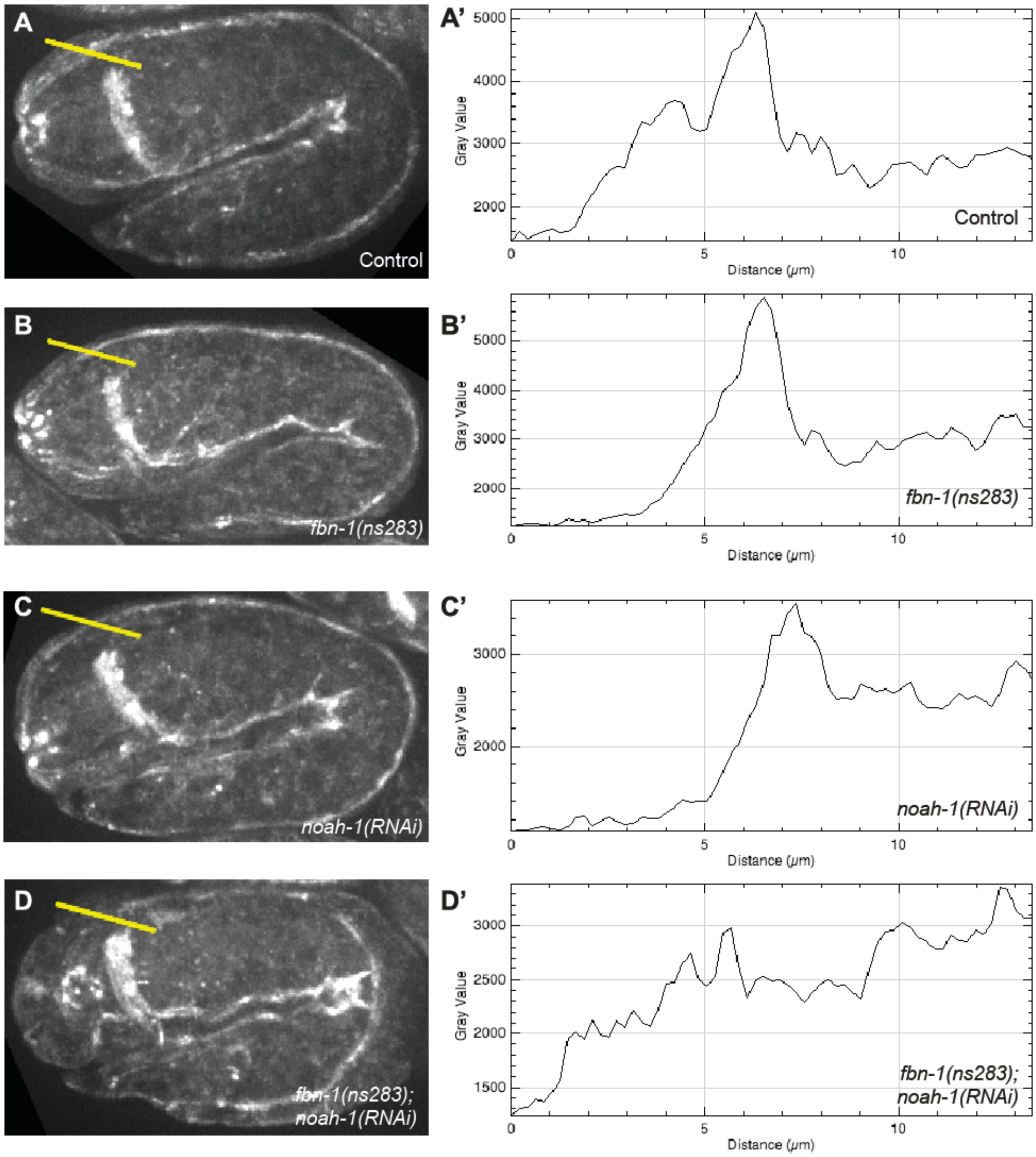

Fig. S7. Loss of GIT-1 localization in embryos depleted for FBN-1 and NOAH-1. Fluorescence pictures and line profiles (yellow lines) of GIT-1::GFP of in control (**A**, **A**'), *fbn-1(ns283)*(**B**, **B'**), *noah-1(RNAi)*(**C**,**C**') and *fbn-1(ns283);noah-1(RNAi)*(**D**,**D**') embryos. Line profile was 3 pixels in width from the outside to the inside of the embryos.

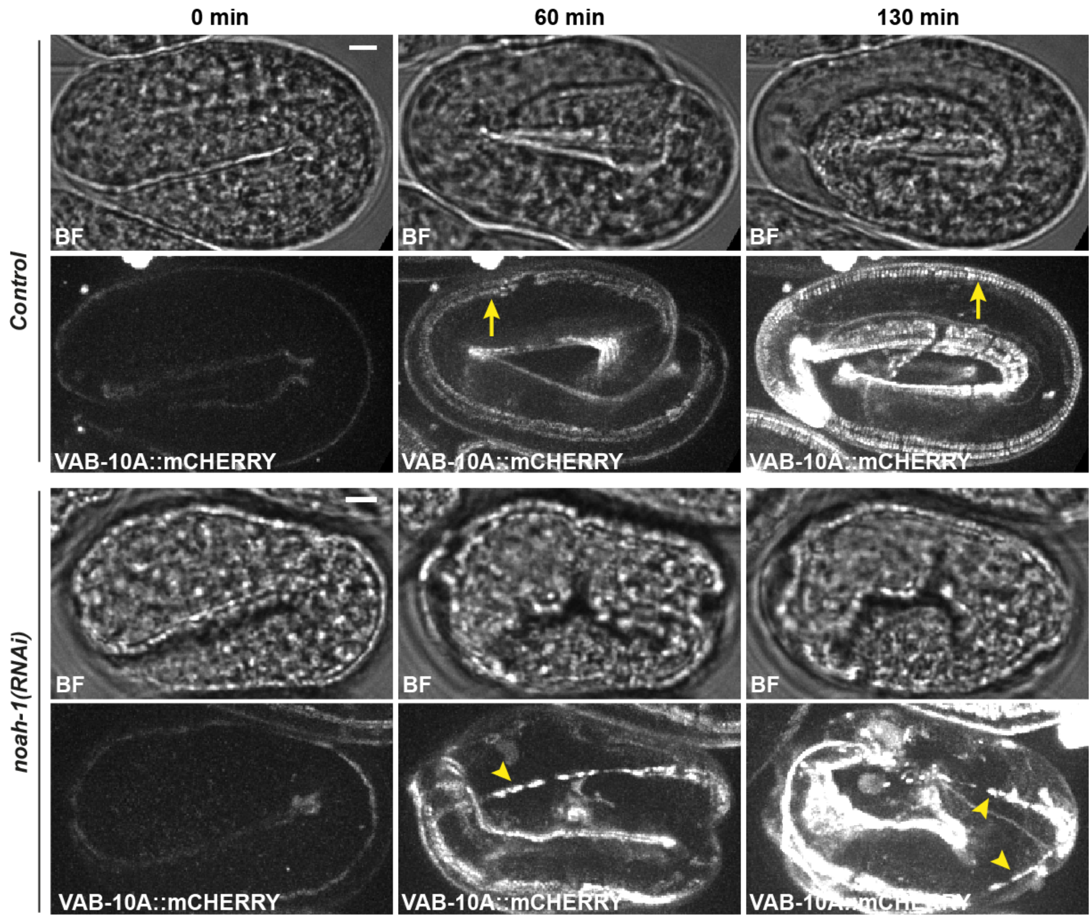

Fig. S8: Rare hemidesmosome defects found in *noah-1(RNAi)* embryos. Comparison of a time-lapse sequence for control and *noah-1(RNAi)* embryos carrying a VAB-10A::mCHERRY(Cter) marker. Row 1 and 3, bright field (BF) pictures; row 2 and 4, Z-projection of fluorescence images of the whole embryo (35 µm). In control embryos, VAB-10A accumulated in four longitudinal stripes of similar width along the AP axis (arrows, N=12/12), whereas in one out of ten *noah-1(RNAi)* imaged embryos, VAB-10A staining became discontinuous and unequal in width (arrowheads). Scale bars, 5 µm.

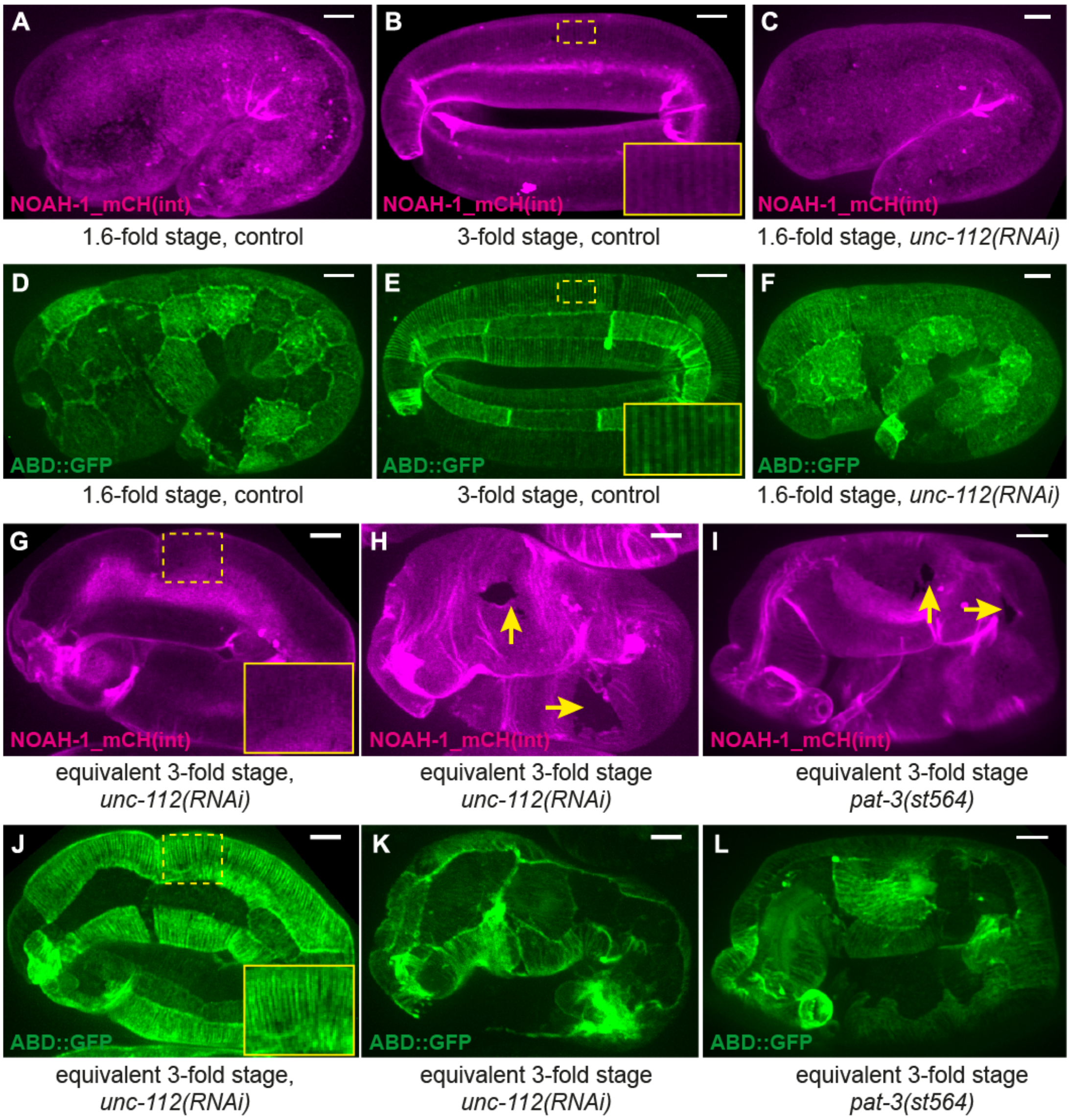

Fig. S9: Muscle contractions affect embryonic sheath remodeling. Embryos carrying NOAH-1_mCH(int) (**A-C**, **G-I**) and ABD::GFP (**D-F**, **J-L**) markers in control (**ABDE**) or UNC-112/Kindlin depleted (**CFGHJK**) or in β-integrin defective *pat-3(st564)* (**IL**) embryos. NOAH-1_mCH(int) localization was similar to wild-type before the 2-fold stage (**AC**), but the circumferential stripes in epidermal DV cells at equivalent 3-fold stage were not seen (**BEGJ**). (**HI**) Holes in the embryonic sheath (arrows) were present. All panels are Z-projections; inserts show the magnified view of the dashed yellow rectangle; scale bar, 5 μm.

Development • Supplementary information

Supplementary tables

Table S1: Genes used in the RNAi screen.

Click here to Download Table S1

Table S2: Embryonic lethality of *noah-1(ok1587)* and *noah-2(ok3197)* mutants, and of the CRISPR knock-in strains NOAH-1_mCH(int) and NOAH-2_GFP(int). The number of surviving larvae with and without balancers, *dpy-5(e61) mcIs50* for *noah-1(ok1587)* and *nT1 [qIs51] (IV;V)* for *noah-2(ok3197),* were scored. ND, not determined. *: this percentage of surviving larvae corresponds to the recombination rate 2/100 of between *dpy-5(e61)* and *mcIs50* previously determined.

Mother genotype	Number of embryos	% dead embryos	% larvae with balancer	% larvae without balancer
noah-1(ok1587)/dpy-5(e61) mcIs50[lin-26p::ABDvab-10::gfp+myo-2p::gfp] I	237	23.6	75.1	1.3*
noah-2(ok3197) IV/nT1 [qIs51] (IV;V)	428	69.9	30.1	0.00
noah-1(mc68[noah-1_mCH(int)]) I	421	1.4	ND	ND
noah-2(mc93[noah-2_flag_gfp(int)]) IV	449	0.1	ND	ND

Development • Supplementary information

Table S3: Embryonic lethality and phenotype scoring of different genetic backgrounds. [a]: phenotype scoring using DIC time-lapse. [b]: embryonic lethality scoring on plates. ND, not determined.

	Number of embryos	Arrest before elongation (%)	Arrest and rupturing around 2-fold (%)	Arrest and rupturing around 3-fold (%)	Normal elongation or hatch (%)	Total embryonic lethality (%)
noah-1 (RNAi)[a]	39	5.1	0.0	92.3	2.6	97.4
fbn-1(ns67)[a]	87	1.1	0.0	0.0	98.9	1.1
fbn-1(ns283)[a]	44	0.0	0.0	0.0	100.0	0.0
fbn-1(tm290 [a]	43	0.0	0.0	0.0	100.0	0.0
fbn-1(ns67); noah-1(RNAi)[a]	37	0.0	54.1	45.9	0.0	100.0
fbn-1(ns283); noah-1(RNAi)[a]	44	2.3	95.5	2.3	0.0	100.0
fbn-1(tm290); noah-1(RNAi)[a]	19	0.0	100.0	0.0	0.0	100.0
noah-2(RNAi)[a]	40	0.0	0.0	90.0	10.0	90.0
fbn-1(ns67); noah-2(RNAi)[a]	42	0.0	9.5	90.5	0.0	100.0
fbn-1(ns283); noah-2(RNAi)[a]	41	0.0	100.0	0.0	0.0	100.0
let-4(RNAi)[a]	52	0.0	0.0	11.5	88.5	11.5
fbn-1(ns67); let-4(RNAi)[a]	81	2.5	3.7	17.3	76.5	23.5
fbn-1(ns283); let-4(RNAi)[a]	45	4.4	0.0	95.6	0.0	100.0
let-4(RNAi) [b]	724	N.D	N.D	N.D	N.D	2.6
sym-1::GFP(Cter)[b]	263	N.D	N.D	N.D	N.D	0.4
sym-1(mn601); let-4(RNAi)[b]	522	N.D	N.D	N.D	N.D	95.4
sym-::GFP(Cter); let-4(RNAi)[b]	330	N.D	N.D	N.D	N.D	63.6

Table S4: *C. elegans* strains used in this study. (a): strains obtained from the CGC. (b): strains kindly provided by D. Fay (Kelley *et al.*, 2015). (c): strains created in this study.

Click here to Download Table S4

Development • Supplementary information

Movies

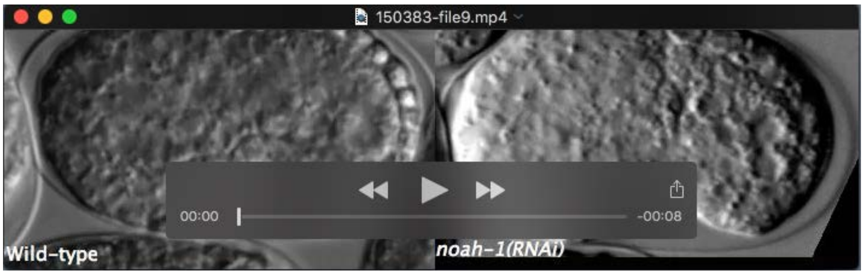

Movie 1: DIC time-lapse of a wild-type (left) and a typical *noah-1(RNAi)* (right) embryo starting at the beginning of elongation. The *noah-1(RNAi)* embryo starts showing some abnormal signs, a small bulge (arrow) and some extra-embryonic material (arrowhead), at 270 min. Another bulge (arrow at 290 min) and more abundant extra-embryonic material (arrowheads after 270 min) were observed at later time

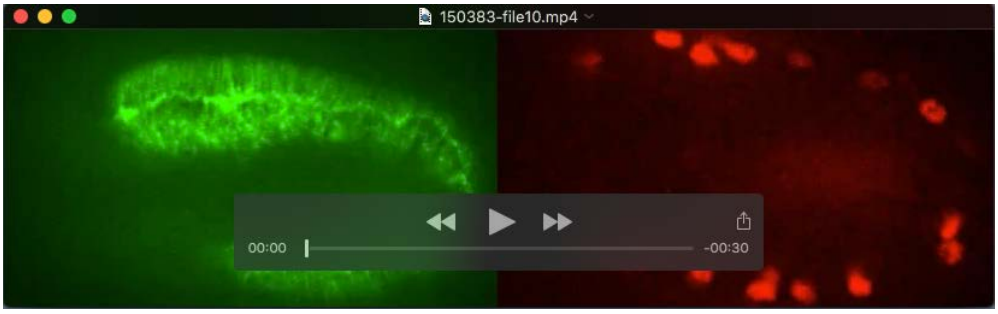

Movie 2: Time-lapse of a wild-type embryo, visualized with an actin marker (LIFEACT::GFP, left) expressed in the epidermis and a nuclear marker (HIS-24::mCHERRY, right) expressed in muscles, showing displacement of actin filaments and muscle nuclei due to muscle contractions (Zhang *et al.*,2011), and rolling movements. The embryo is 10 min past the 2-fold stage (80 min after ventral enclosure). The movie on the right was started around 20 s after the movie on the left.

Development • Supplementary information

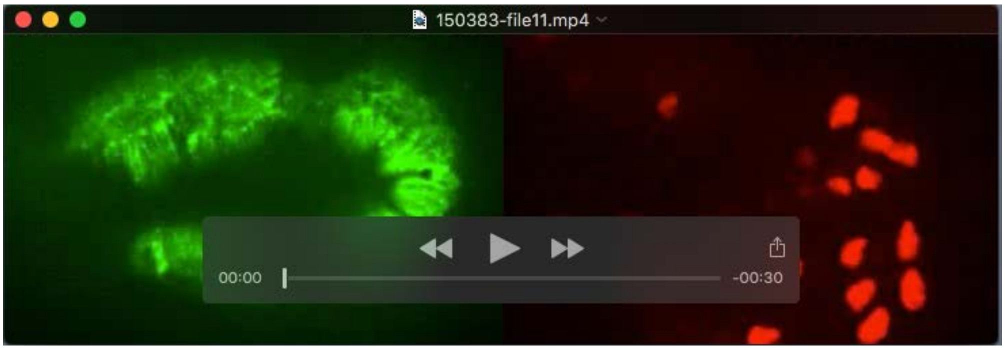

Movie 3: Time-lapse of a *fbn-1(ns283); noah-1(RNAi)* embryo, visualized with an actin marker (LIFEACT::GFP, left) expressed in the epidermis and a nuclear marker (HIS-24:: mCHERRY, right) expressed in muscles, showing reduced twitching and no rolling compared to wild-type control. The embryo is at the same stage as in movie 1. The movie on the right was started around 20 s after the movie on the left.

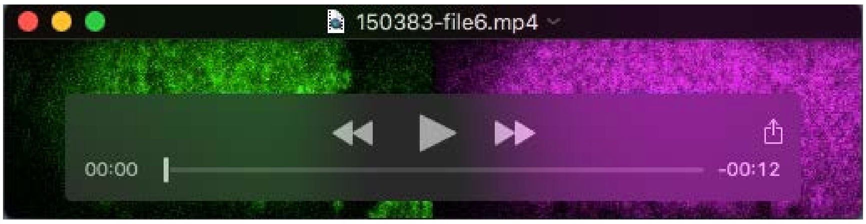

Movie 4: Example of laser ablation in the seam cell H1 of a 1.7-fold control embryo, visualized with actin (ABD::GFP) and embryonic sheath (NOAH-1_mCH(int)) markers. The cyan line at the beginning of the movie shows the cut line of 5 μm in length.

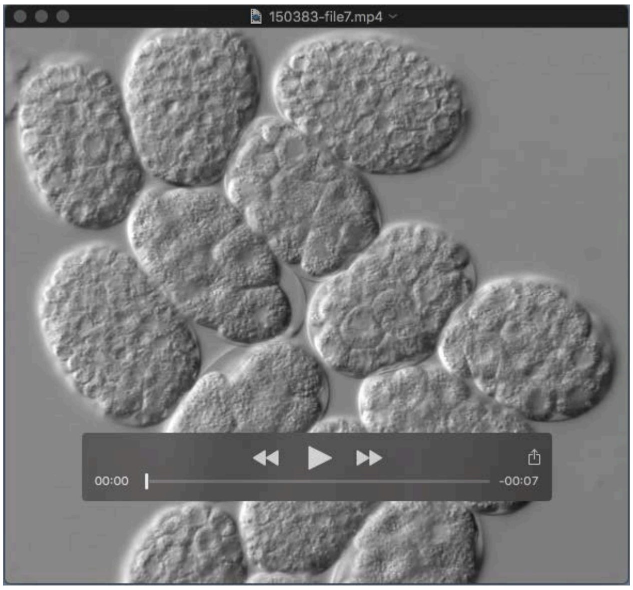

Movie 5: Time-lapse of *let-502(sb118ts); pak-1(ok448); noah-1(RNAi)* embryos recorded at the restrictive temperature 25.5°C, showing inhibition of elongation and absence of rupture.

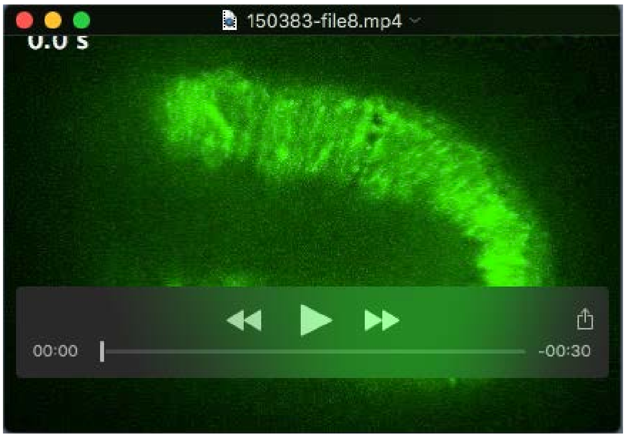

Movie 6: Time-lapse of *pak-1(ok448); noah-1(RNAi)* embryos at a late elongation stage, visualized with LIFEACT::GFP, showing normal muscle twitching and rolling. Time is in hour:minute.
